# Glucocorticoid-induced microRNA-378 signaling mediates the progression of pancreatic cancer by enhancing autophagy

**DOI:** 10.1038/s41419-022-05503-3

**Published:** 2022-12-19

**Authors:** Li Liu, Shanshan Han, Xi Xiao, Xuefeng An, Jury Gladkich, Ulf Hinz, Stefan Hillmer, Torsten Hoppe-Tichy, Yi Xu, Michael Schaefer, Oliver Strobel, Ingrid Herr

**Affiliations:** 1 Section Surgical Research, Department of General, Visceral and Transplantation Surgery, University of Heidelberg, Heidelberg, Germany; 2 Department of General, Visceral and Transplantation Surgery, Ruprecht Karls University of Heidelberg, Medical Faculty, Heidelberg, Germany; 3 Electron Microscopy Core Facility, University of Heidelberg, Heidelberg, Germany; 4 Clinic Pharmacy, University of Heidelberg, Heidelberg, Germany; 5 School of Chemical and Environmental Engineering, Shanghai Institute of Technology, Shanghai, China; 6 Department of General Surgery, University of Vienna, Vienna, Austria

**Keywords:** Gastrointestinal cancer, Translational research, Preclinical research, Transcription

## Abstract

Glucocorticoids (GCs) are widely used in tumor therapy to reduce tumor growth, inflammation, edema, and other side effects. Controversially, GCs may also cause the progression of highly aggressive pancreatic ductal adenocarcinoma (PDAC). Because microRNA (miR) and autophagy signaling support the invasive growth of PDAC, we asked whether these mechanisms may be targeted by GCs. Six established human PDAC cell lines, tissue from patients who received GC medication (*n* = 35) prior to surgery, or not (*n* = 35), and tumor xenografts were examined by RT‒qPCR, transmission electron microscopy (TEM), monodansylcadaverine (MDC) staining, immunohistochemistry, in situ hybridization, gene array and Kaplan‒Meier analysis with bioinformatics, and MTT, western blot, colony, spheroid, migration, and invasion assays. We found that various GCs, including dexamethasone (DEX), induced typical features of macroautophagy with the appearance of autolysosomes, enhanced LC3-II, decreased SQSTM1/p62 expression and induced epithelial-mesenchymal transition (EMT) and gemcitabine resistance. The GC receptor (GR) antagonist mifepristone (RU486) counteracted DEX-induced autophagy features, suggesting that the GC-GR complex is involved in the induction of autophagy. The autophagy-related miR-378i and miR-378a-3p were selected as the top upregulated candidates, and their high expression in PDAC patient tissue correlated with low survival. siRNA-mediated downregulation of miR-378 inhibited DEX-induced autophagy, and tumor progression. Bioinformatics confirmed the contribution of miR-378 to the regulation of signaling networks involved in GC-induced autophagy and tumor progression. The construction of a molecular docking model revealed stable binding of miR-378 to the DEX-GR complex, suggesting direct regulation. These substantial, novel, in-depth data reveal that GCs favor autophagy-mediated cancer progression by inducing miR-378 and GR binding and implicate GR and miR-378 as new therapeutic targets.

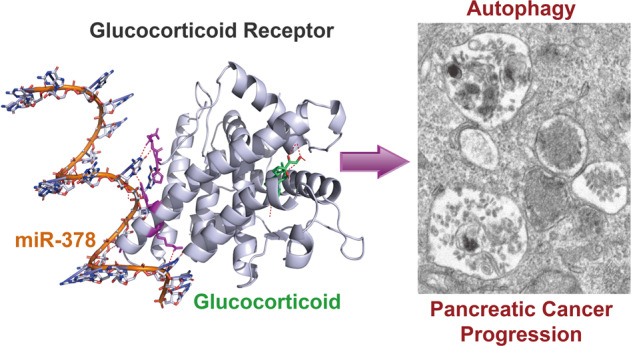

## Introduction

Pancreatic ductal adenocarcinoma (PDAC) grows mainly on chronically inflamed pancreatic tissue, and its poor prognosis is particularly associated with early metastasis, late diagnosis, and severe cachexia [1, 2].

In epithelial tumors such as PDAC, GC medications are prescribed to control the side effects of chemotherapy and tumor growth [3], including cachexia, pain [2], inflammation [4], and tumor-related edema [5], and to increase appetite and general well-being. These positive GC effects are offset by numerous reports describing GC-induced therapeutic resistance and tumor progression in epithelial cancers [3, 6–9]. Most importantly, several clinical studies have demonstrated an enhanced risk of tumor progression and metastasis upon the intake of GC medication in patients with ovarian, breast, lung, glioblastoma, oral squamous cell, skin and bladder carcinomas, various other solid cancers, and non-Hodgkin’s lymphoma [10–19].

Previous reports suggest that GCs induce autophagy in chondrocytes and lymphoma cells, which then protects them from GC-induced apoptosis [20, 21]. However, mechanistic information and the relevance of these findings for epithelial cancer are not yet available. Though, putative GC-induced autophagy in PDAC would be a highly relevant new therapeutic target, as increased levels of autophagy have been associated with poor prognosis in PDAC patients [22, 23]. Autophagy is an important survival mechanism that supplies nutrients, particularly amino acids, for new protein synthesis by degrading proteins and lipids under nutritional deprivation and cellular stress conditions [24]. Therefore, autophagy enables the survival of tumor cells despite chemotherapy and a hypoxic tumor microenvironment [25]. Autophagy signaling starts with the formation of double-membraned vesicles called autophagosomes, which then fuse with lysosomes to form autolysosomes, in which cellular waste is degraded [26, 27]. Key players are autophagy-related (ATG) proteins, especially Atg4B and Atg12, the conversion of the cytosolic, soluble LC3-I protein to LC3-II, which then binds to membranes of autophagosomes [28], and the autophagic substrate SQSTM1/p62, the abundance of which reflects autophagic flux [29]. In addition, monodansylcadaverine (MDC) is an autofluorescent and specific marker that is incorporated into the membranes of autophagic vacuoles and therefore serves for their detection [30]. A growing body of evidence suggests that autophagy favors the growth and progression of malignant tumors, particularly during epithelial to mesenchymal transition (EMT) [31]. Conversely, deficient autophagy mechanisms can limit the proliferation, dissemination, and metastatic potential of malignant cells. Increased autophagic flux is typically found in advanced cancer and is associated with a metastatic phenotype and poor prognosis [32]. We recently found that GCs induce EMT and cancer stem cell features in experimental PDAC and patient tissues by activating TGF-β expression and GR and JNK/AP-1 signaling [33]. Our recent results also reveal that microRNA-132 is inhibited by DEX-induced methylation, which contributes to TGF-β-driven PDAC progression [34].

MicroRNAs (miRs) are highly conserved, noncoding RNAs generally consisting of 18–24 nucleotides, but sometimes hundreds of nucleotides, which base-pair with conserved sequences in the 3′ untranslated regions (3′UTRs) of target messenger RNAs (mRNAs). miRs control gene expression posttranscriptionally by inhibiting translation or by degrading a target mRNA. Although GC-induced miR signaling in PDAC has only been marginally investigated thus far, a growing body of evidence indicates that GCs induce epigenetic signaling by modulating miR signaling [35–40]. Several studies suggested miRs as key mediators of GC signaling, because miRs are involved in regulation of GC synthesis in the adrenal gland and in modulation of the cellular response to GC signaling [41].

In the present study, we inquired whether GC medication mediates PDAC progression by inducing miRNA-driven autophagy. By the use of established cell lines, mouse xenografts and tissue from PDAC patients who received GC medication prior to surgery and from those who did not, we highlight an important function of miR-378 signaling in GC-GR complex-induced autophagy, which ultimately converges in cancer progression.

## Results

### GCs induce EMT- and autophagy-related marker expression

**Fig. 1:**
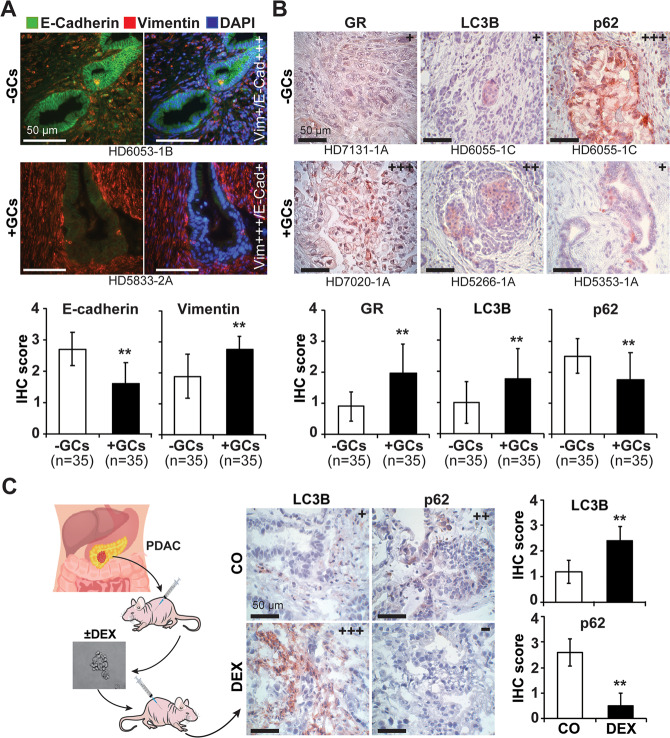
GC treatment is associated with the expression of EMT and autophagy markers. **A** Representative paraffin-embedded PDAC sections derived from patients with preoperative administration of glucocorticoids (+GCs, *n* = 35, compare Supplemental Tables S1 and S2) or without administration (-GCs, *n* = 35) were evaluated by double immunofluorescence staining of the epithelial marker E-cadherin (green) and the mesenchymal marker vimentin (red). The cell nuclei were counterstained with DAPI (blue). Ten randomly chosen fields of each tissue were evaluated in a double-blinded manner under 400× magnification by semiquantitative scoring of the fluorescence intensity and the percentage of positively stained cells. Score 4 (++++): very strong positive fluorescence and very high percentage of positive cells (≥75%); Score 3 (+++): strong positive fluorescence and high percentage of positive cells (≥50%); Score 2 (++): moderate positive fluorescence and medium percentage of positive cells (≥25%); Score 1 (+): weak positive fluorescence and low percentage of positive cells (≤25%); Score 0 (−): no positive fluorescence and no positive cells (0%). The scale bar indicates a distance of 50 µM. **B** The expression levels of GR, LC3B and SQSTM1/P62 were detected by immunohistochemical staining and quantified as described above. **C** Tumor cells were isolated from freshly resected PDAC patient tissue and subcutaneously transplanted into the flanks of immunodeficient mice, followed by subtransplantation until a stably growing xenograft tumor was obtained. The xenografts were resected, and the primary tumor cells were isolated by mincing and filtration, followed by spheroidal culture in vitro. One half of the cells was treated with 1 μM dexamethasone (DEX), and the other half was left untreated (CO). Forty-eight hours later, equal amounts of viable cells were transplanted into the flanks of immunodeficient mice (*n* = 6). Three weeks after transplantation, the xenografts were resected, and the expression of the autophagy markers LC3B and SQSTM1/p62 was detected in xenograft sections by immunohistochemistry under 400× magnification. Representative images are shown. The quantitation was performed as described above, and mean values with standard deviations are given. **P < 0.01.

To evaluate the impact of GCs on autophagy signaling, which is often associated with EMT [31], we stained PDAC tissue from patients who had (*n* = 35) or had not (*n* = 35) taken GCs prior to surgery (Suppl. Tables S1 and S2). Double immunofluorescence staining of EMT markers, detection by fluorescence microscopy, and evaluation of the intensity of positive signals by a scoring system revealed significant upregulation of vimentin and downregulation of E-cadherin expression in tissue from patients who had received GC treatment compared to those who had not, as shown by representative images and the mean values in diagrams (Fig. 1A). Additionally, GC treatment resulted in significantly higher expression of GR, as examined by immunohistochemistry (Fig. 1B). Most importantly, the autophagy marker LC3B was significantly upregulated, and the autophagic flux marker SQSTM1/p62 was significantly downregulated upon GC intake. Induction of autophagy signaling was confirmed by isolation of primary PDAC xenografts from patient tissue by xenotransplantation to immunodeficient mice, resection of the xenografts 3 weeks later, culture as in vitro spheres, in vitro DEX treatment, retransplantation to mice and immunohistochemical detection of autophagy-related marker proteins in xenograft tissue sections 3 weeks later (Fig. 1C).

### DEX induces typical autophagy-related morphological changes

**Fig. 2:**
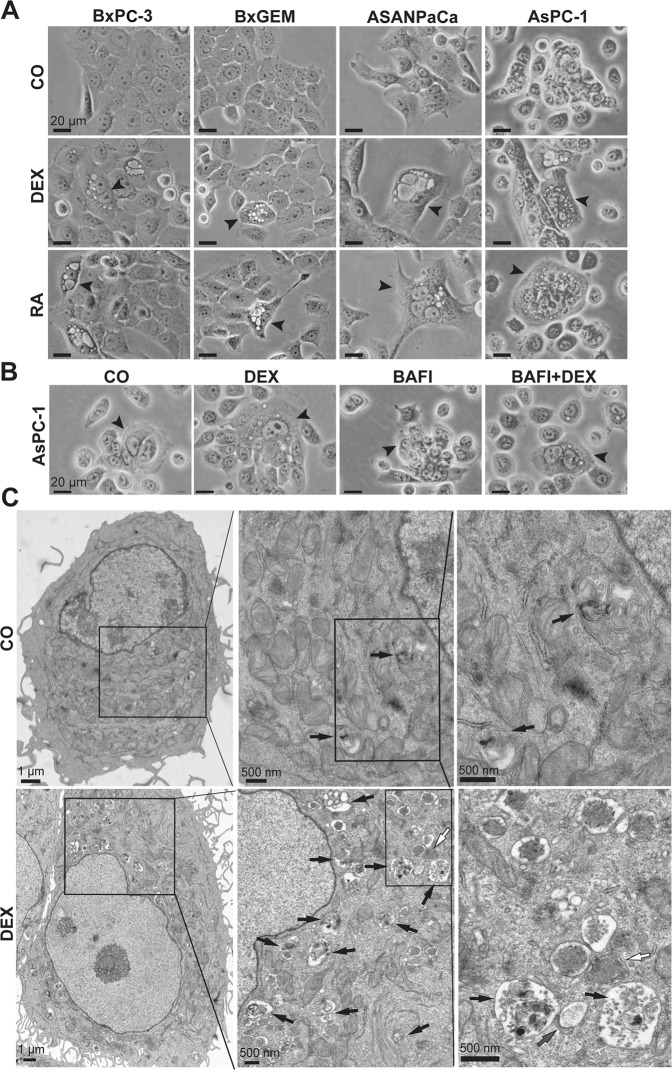
DEX induces vacuole formation typical for autophagy. **A** BxPC-3, gemcitabine-resistant BxGEM, ASAN-PaCa and AsPC-1 cells were cultured in medium containing 1 µM DEX, 20 µM of the autophagy inducer rapamycin (RA), or ethanol vehicle control (CO) for 24 h. Then, the cell morphology was visualized by phase-contrast microscopy under 400× magnification using a Nikon Eclipse TS100 microscope. Black arrows indicate representative cells with vacuoles. **B** AsPC-1 cells were cultured with 1 µM DEX, 10 nM bafilomycin A1 (BAFI), both together (BAFI + DEX), or were treated with ethanol vehicle control alone (CO) for 24 h. The cell morphology was visualized by phase-contrast microscopy under 400× magnification using a Nikon Eclipse TS100 microscope. Black arrows indicate representative cells with vacuoles. **C** AsPC-1 cells were treated as described above, and the formation of autophagic vesicles was evaluated by electron microscopy. White arrows: autophagosomes; black arrows: autolysosomes; gray arrows: isolation membranes. The sections were viewed using a Zeiss EM10 electron microscope at 80 kV and 6000× magnification. One representative image each of control and DEX-treated cells is shown. Enlargements of an area are marked by boxes, and the scale bars of 1 µM and 500 µM are shown.

To investigate cellular morphology upon GC treatment, the established PDAC cell lines were cultured in medium containing 1 µM DEX or 20 µM of the autophagy inducer rapamycin, whereas control cells were treated with ethanol vehicle alone. Twenty-four hours later, the cells were evaluated by microscopy. Increased vacuole formation occurred upon DEX or rapamycin treatment and suggested the induction of autophagy (Fig. 2A). This was confirmed by coincubation of AsPC-1 cells with DEX in the presence of the autophagy inhibitor bafilomycin A1, which prevented vacuole formation (Fig. 2B). To further corroborate these findings, transmission electron microscopy (TEM) was performed and revealed that DEX induced the accumulation of autophagosomes and autolysosomes (Fig. 2C).

### DEX induces autophagy via activation of GR

**Fig. 3:**
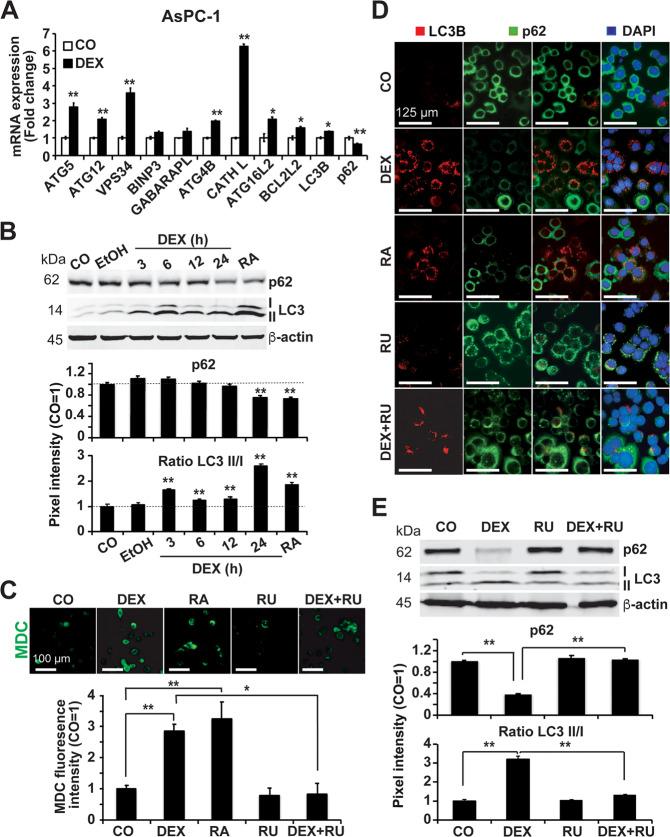
GR is involved in DEX-induced formation of autophagic vacuoles. **A** AsPC-1 cells were treated with 1 µM DEX or with ethanol vehicle control (CO), followed by harvesting of the total RNA 24 h later. RT‒qPCR was performed using the specific autophagy-related primers ATG5, ATG12, VPS34, BINP3, GABARAPL, ATG4B, CATHEPSIN L (CATH L), ATG16L2, BCL2L2, LC3B, and SQSTM1/p62 (p62). Gene expression was normalized to that of GAPDH, and the relative mRNA expression is shown as the fold change. The data are presented as the mean values with standard deviations. *P < 0.05, **P < 0.01 compared to the control group. **B** AsPC-1 cells were incubated in medium with 1 µM DEX or 20 µM rapamycin (RA), left untreated (CO) or treated with ethanol vehicle control (EtOH) for 24 h or the indicated time points. The expression of LC3-I, LC3-II and SQSTM1/p62 (p62) was detected by western blot analysis. β-actin served as a loading control. The protein sizes in kilodalton (kDa) are given on the left. The diagrams depict the pixel intensity of the bands, which was measured using Image Studio after normalization to ®-actin. The pixel intensity of the control group was set to 1. **P < 0.01 compared to the control group. The black dotted line represents 100%. **C** MDC staining of acidic vacuoles: AsPC-1 cells were cultured in medium with 1 µM DEX or 20 µM rapamycin (RA), 1 µM of the GR inhibitor mifepristone/RU486 (RU), DEX plus mifepristone/RU486 (DEX + RU), or treated with ethanol vehicle control alone (CO) for 24 h. Autophagic vacuoles were labeled with the specific autofluorescence marker MDC (green), followed by immediate examination by fluorescence microscopy under 200× magnification. The scale bar indicates 100 µm. The MDC fluorescence intensity was quantified by ImageJ, and the density of the control was set to 1. *P < 0.05, **P < 0.01. **D** AsPC-1 cells were treated as described above. Autophagy-related protein expression of LC3B (red) and SQSTM1/p62 (p62, green) was detected by double-fluorescence staining under 1000× magnification. The cell nuclei were counterstained with DAPI (blue). The scale bar indicates 125 µm. **E** AsPC-1 cells were cultured in medium with 1 µM DEX, 1 µM mifepristone/RU486 (RU), both together (DEX + RU), or with ethanol vehicle control alone (CO) for 24 h. The expression of LC3-I, LC3-II and SQSTM1/p62 (p62) was detected by western blot analysis. β-Actin served as a loading control. The protein sizes in kilodalton (kDa) are given on the left. The pixel intensity of the bands was detected by the use of ImageJ, and the means and standard deviations are shown in the diagram. *P < 0.05, **P < 0.01.

We next examined the expression of autophagy genes by qRT‒PCR following treatment of PDAC cells with DEX and harvesting of the mRNA 24 h later. We found increased expression of the autophagy-related genes LC3B, ATG5, ATG12, ATG4B, VPS34, CATHEPSIN L, BCL2L2 and ATG16L2, along with decreased expression of SQSTM1/p62 (Fig. 3A, Suppl. Fig. S1A). These results were confirmed by western blot analysis, which demonstrated enhanced LC3-II expression along with reduced SQSTM1/p62 protein expression (Fig. 3B and Suppl. Figs. S1B and S2). The autophagy inducer rapamycin was used as a positive control and confirmed DEX-induced autophagy. To detect the formation of autophagic vacuoles, we stained autophagic vesicles with the specific autofluorescent marker MDC. DEX increased the number of MDC-labeled autophagic vacuoles and overall MDC fluorescence, similar to rapamycin (Fig. 3C and Suppl. Fig. S1C and S3A). Inhibition of GR signaling by mifepristone/RU486 prevented DEX-induced formation of autophagic vacuoles, suggesting that GR signaling is required for the formation of autophagosomes. These results were confirmed by immunofluorescence staining of LC3-II and SQSTM1/p62 expression following DEX treatment in the presence or absence of rapamycin or RU486 (Fig. 3D and Suppl. Fig. S1D and S3B). Finally, western blot analysis and quantification of pixel intensities strengthened these results (Fig. 3E and Suppl. Fig. S3C).

### Several clinically applied GCs promote autophagy and therapy resistance

**Fig. 4:**
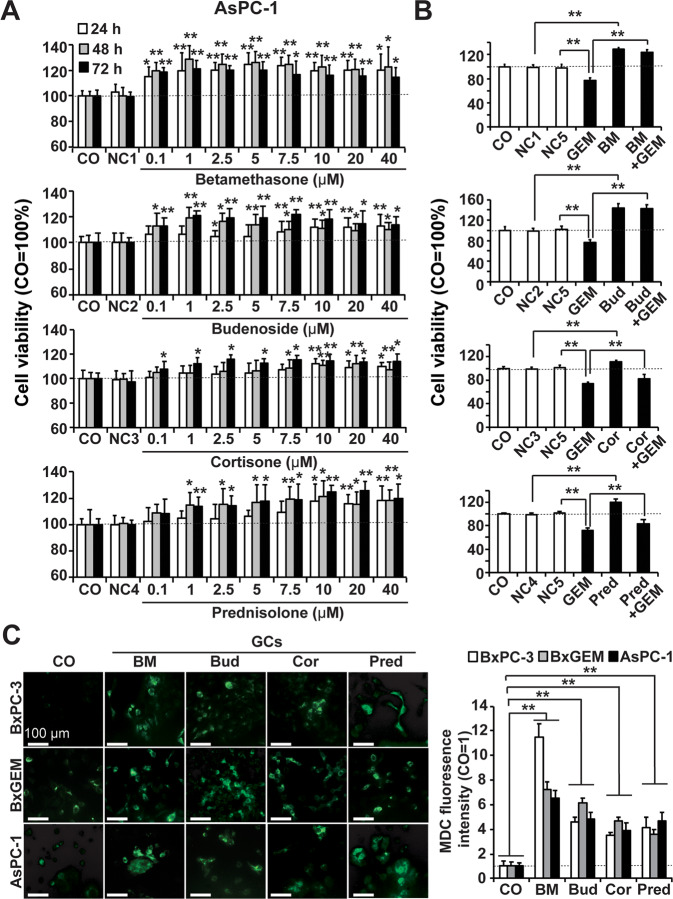
Various GCs enhance viability and induce gemcitabine resistance and autophagosome formation. **A** AsPC-1 cells were treated with the GCs betamethasone, budesonide, cortisone, and prednisone at the concentrations indicated or with vehicle controls alone (NC1: DMSO 1:50,000; NC2: DMSO: 1:100,000; NC3: methanol: 1:25,000; NC4: DMSO: 1:100,000), followed by MTT assay 24, 48 and 72 h later. The controls of each timepoint were set to 100%. *P < 0.05, **P < 0.01 compared to the negative control (NC). **B** AsPC-1 cells were cultured in medium containing 1 µM betamethasone (BM), 1 µM budesonide (Bud), 10 µM cortisone (Cor), or 10 µM prednisone (Pred) or were treated with vehicle alone as described above (NC5: PBS: 1:126,000) or were left untreated (CO). Then, 48 h later, 50 nM gemcitabine (GEM) was added to untreated and GC-treated cells as indicated for another 48 h. The viability was measured by MTT assay. The percentage of viable cells in the control groups was set to 100%. *P < 0.05, **P < 0.01. **C** MDC staining of acidic vacuoles: BxPC-3, BxGEM and AsPC-1 cells were cultured in the presence of different GCs as indicated above for 24 h or were left untreated (CO). The cells were labeled with MDC (green) followed by immediate monitoring under a fluorescence microscope at 200× magnification. The MDC fluorescence intensity was evaluated by ImageJ, and the density in the control was set to 1. The control line is marked by a black dotted line. The means with standard deviations are shown. *P < 0.05, **P < 0.01 compared to the control group.

To assess the effect of a variety of clinically used GCs, PDAC cells were treated with betamethasone, budesonide, cortisone and prednisolone at concentrations of 0.1, 1, 2.5, 5, 7.5, 10, 20, and 40 µM for 24, 48, and 72 h. Compared to untreated or vehicle-treated controls, all GC species significantly enhanced the cell viability at the lowest concentrations and earliest time points, as detected by MTT assay (Fig. 4A and Suppl. Fig. S4A). The most effective concentrations were 1 μM betamethasone, 1 μM budesonide, 10 μM cortisone and 10 μM prednisone. Therefore, this selection was chosen for the evaluation of GC-induced gemcitabine resistance. BxPC-3 and AsPC-1 cells were pretreated with GCs for 48 h, followed by gemcitabine cotreatment for another 48 h, whereas the controls were left untreated or were treated with GCs or gemcitabine alone. Whereas gemcitabine alone significantly reduced viability, coincubation with GCs significantly counteracted this effect (Fig. 4B and Suppl. Fig. S4B). MDC staining and fluorescence microscopy confirmed these results (Fig. 4C). We concluded that not only DEX, as we described previously [33], but GCs in general induce autophagy and evoke gemcitabine resistance.

### GCs regulate miR expression

**Fig. 5:**
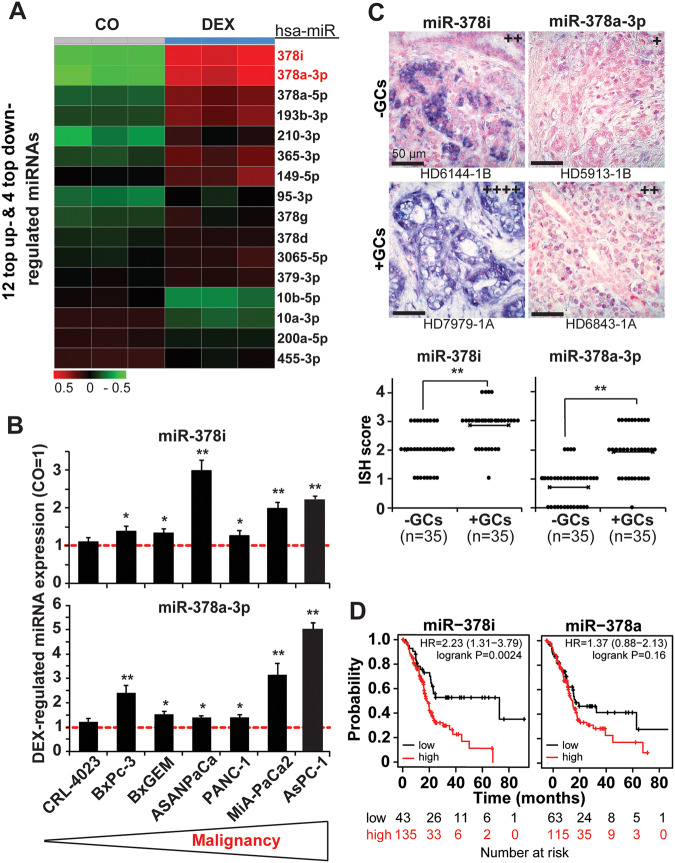
DEX induces autophagy-related miRNA expression, and the top miR-378 predicts patient survival. **A** AsPC-1 cells were cultured in medium containing 1 µM DEX or were treated with ethanol vehicle control (CO) for 24 h. The RNA was harvested using the Qiagen miRNeasy Mini Isolation Kit. The miRNA expression was analyzed by an Agilent miRNA Microarray (Release 19.0). The heatmap presents the top 16 most significantly differentially upregulated (12) and downregulated (4) miRs. Red: upregulation; green: downregulation within a scale from 0.5 to −0.5. **B** BxPC-3, BxGEM, AsPC-1, ASAN-PaCa, PANC-1, and AsPC-1 cells and nonmalignant immortalized pancreatic duct cells (CRL-4023) were incubated in medium containing 1 µM DEX or in ethanol vehicle control. Then, 24 h later, the miRNA was isolated, and RT‒qPCR was performed using specific primers for the detection of the most significant miR-378i and miR-378a-3p. The expression levels were normalized to RNU44, and the mean fold changes of the control cells were set to 1. The means and standard deviations are shown. The red dotted line indicates the control value of 1. *P < 0.05, **P < 0.01 compared to the control group. **C** DIG-labeled hsa-miR-378i and hsa-miR-378a-3p probes were hybridized to paraffin-embedded human PDAC tissue. Representative paraffin-embedded sections (HD 6144-1B, HD 7979-1 A, HD 5913-1B, HD 6843-1 A) derived from patients who received GCs prior to surgery (+GCs, *n* = 35) or not (-GCs, *n* = 35) were evaluated as described in Fig. 1. The miR expression levels were quantified by in situ hybridization. The cell nuclei were stained with Fast Red. The dark purple miR-positive signal was evaluated in 10 randomly chosen fields of each tissue in a blinded manner by two examiners with expertize in PDAC pathology under 400× magnification. The scoring system described in Fig. 1 was used for quantification. The quantitative data are presented as the mean values with standard deviations. **P < 0.01. **D** Overall survival data of PDAC patients relative to the expression of hsa-miR-378i or hsa-miR-378a-3p were obtained from the Kaplan‒Meier plotter online platform. A log-rank *P* < 0.05 was considered to indicate a statistically significant difference. Expression above the median: red line; expression below the median: black line. The hazard ratio (HR) indicates the difference between the red and black lines. HR = 1: no difference. HR < 1: high miR expression is associated with longer survival. HR > 1: high miR expression is associated with shorter survival.

Based on a recently suggested role of miRs in GC-induced EMT signaling [34], we next evaluated the impact of miR signaling in GC-induced autophagy. AsPC-1 cells were treated with DEX or vehicle alone as the control, followed by harvesting RNA 24 h later and miRNA array analysis. Based on bioinformatics evaluation, 12 miRs were most significantly upregulated, and 4 miRs were most significantly downregulated, with *P* < 0.05, as shown in a heatmap (Fig. 5A). From these miR candidates, we selected the two most significantly upregulated, miR378i and miR378a-3p, and confirmed the DEX-induced expression pattern in diverse PDAC cell lines by RT‒qPCR (Fig. 5B). Compared to PDAC cells, the upregulation was lowest in the nonmalignant immortalized pancreatic duct cell line CRL-4023, suggesting that high expression of miR-378i and miR-378a-3p is related to malignancy. This assumption was confirmed by recent publications, which demonstrated the involvement of miR-378 in the promotion of autophagy, stemness, and chemoresistance (Suppl. Table S3). To highlight the clinical relevance, the expression of miR-378i and miR-378a-3p was evaluated in tissue from PDAC patients who received GC medication prior to surgery (*n* = 35) or not (*n* = 35) by in situ hybridization. Representative images and evaluation of the expression levels by a scoring system confirmed higher expression of miR-378i and miR-378a-3p in response to GC medication (Fig. 5C). Using the online database Kaplan‒Meier plotter, we even found a correlation of high miR-378i and miR-378a-3p expression and shorter overall survival of PDAC patients as compared to PDAC patients with low expression of these miRs (Fig. 5D).

### miR-378 is involved in DEX-induced autophagy, EMT, and stemness

**Fig. 6:**
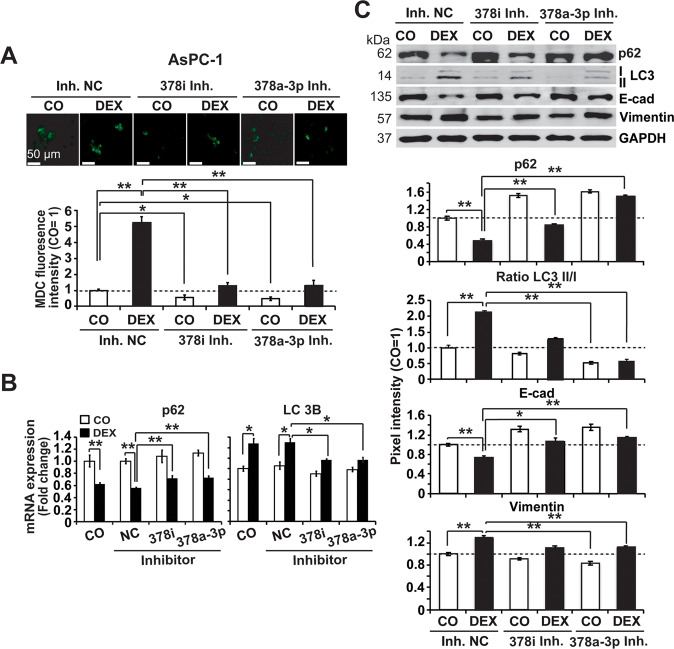
miR-378 inhibits DEX-induced autophagy. **A** AsPC-1 cells were lipotransfected with miR-378i inhibitor (100 nM), miR-378a-3p inhibitor (100 nM) or a noncoding miR inhibitor control (Inh. NC, 100 nM) and cotransfected with firefly luciferase (0.25 ng/μl), which served as a control for equal conditions. Twenty-four hours later, the cells were treated with 1 µM DEX or were left untreated as a control (CO). Then, 24 h later, autophagic vacuoles were labeled with the specific autofluorescence marker MDC (green), followed by immediate examination by fluorescence microscopy under 200× magnification. The scale bar indicates 50 µm. The MDC fluorescence intensity was quantified by ImageJ, and the density of the control was set to 1. *P < 0.05, **P < 0.01. **B** AsPC-1 cells were treated with miRs as described above, followed by harvesting of the total RNA 24 h later. The expression of the autophagy markers LC3B and SQSTM1/p62 (p62) was detected by RT‒PCR using specific primers. Gene expression was normalized to that of GAPDH, and the relative mRNA expression is shown as the fold change. The mean values with standard deviations are shown. *P < 0.05, **P < 0.01. **C** AsPC-1 cells were transfected as described above, and 24 h later, the expression of the autophagy markers SQSTM1/p62 (p62) and LC3-I + LC3-II, and of the EMT markers E-cadherin (E-cad) and vimentin, was detected by western blot analysis. GAPDH served as a loading control. The protein sizes in kilodaltons (kDa) are given on the left. The pixel intensities of the bands of at least three independent experiments were measured using Image Studio, and the intensities were normalized to GAPDH. The pixel intensities of the controls were set to 1 and are represented by the black dotted line. **P < 0.01.

To investigate the effect of miR-378 on autophagy, we lipotransfected inhibitors of miR-378i and miR-378a-3p along with nonspecific control inhibitors in the presence or absence of DEX and evaluated the presence of autophagic vacuoles by MDC staining. This resulted in a strong reduction in basal and DEX-induced MDC fluorescence upon inhibition of miR-378i and miR-378a-3p compared to the nonspecific miRNA control (Fig. 6A and Suppl. Fig. S5). Accordingly, the inhibition of miR-378i and miR-378a-3p significantly prevented DEX-mediated inhibition of SQSTM1/p62 expression or induction of LC3B expression, as evaluated by RT‒qPCR analysis (Fig. 6B). This was confirmed by western blot analysis, as shown by representative blots and diagrams with the means and standard deviations (Fig. 6C, compare with Suppl. Fig. S2). In addition, inhibition of miR-378i and miR-378a-3p partially reversed DEX-mediated inhibition of E-cadherin and induction of vimentin. Likewise, inhibition of these miRs significantly inhibited basal and DEX-induced plating efficiency, spheroid formation, migration and invasion, as found by evaluation of colony and spheroid formation and scratch and Transwell invasion assays (Suppl. Fig. S6A–D).

### Bioinformatic docking simulation reveals stable binding of miR-378 to the DEX-GR complex

**Fig. 7:**
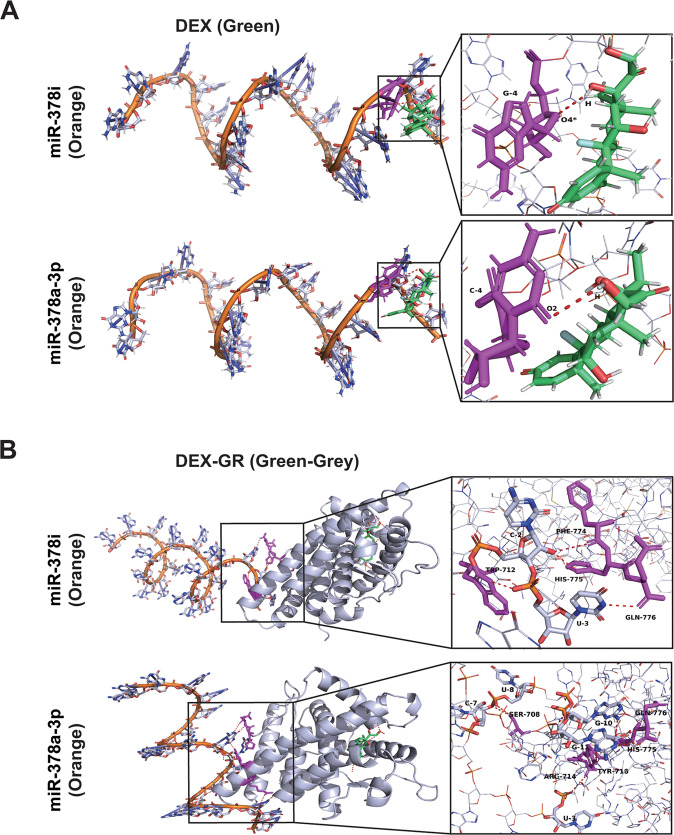
Bioinformatic molecular docking simulation predicts binding of miR-378 to DEX and GR. **A** Molecular docking simulation of miR-378i and miR-378-3p to DEX was performed using YASARA Macro software, and the results were visualized by PyMOL 2.5 software. Green: DEX; Orange: miR-378; Violet: Binding sites of miR-378i and miR-378a-3p. Oxygen atom 4: (O4); Guanine 4: (G-4), H atom: (H); Oxygen atom 2: (O2); Cytosine 4: (C-4). Hydrogen bonds are marked by dotted lines. **B** Interactions between DEX-GR complexes were calculated by the use of the online ZDOCK server, visualized by PyMOL 2.5 and optimized by YASARA Macro software. The obtained model of the DEX-GR complex (green‒gray) was used for detection of putative binding sites with miR-378 (orange) by the use of the ZDOCK server and visualization by PyMOL software. Hydrogen bonds are marked by dotted lines. G guanine, C cytosine, U uracil, O oxygen atom, TRP tryptophan, HIS histidine, PHE phenylalanine, GLN glutamine, SER serine, AGR arginine, TYR tyrosine.

To highlight the mechanisms of the molecular interaction between GCs and miR-378, an online molecular docking analysis based on hydrogen bonds was performed. To simulate putative binding between hydrogen bonds of DEX-GR complexes with miR-378a-3p and miR-378i, respective model graphs were constructed as shown (Fig. 7A). Likewise, molecular docking models were constructed for the detection of putative binding between DEX and the top miR candidates (Fig. 7B). Based on an enhanced number of H-bonds (Suppl. Table S4) and the fact that H-bonds are instrumental in ligand‒receptor binding [42], our docking analysis suggests that the DEX-GR complex binds more stably to miR-378 than DEX alone. These results highlight the regulation of miR-378 by the GC-GR complex and suggest that GR signaling is necessary for the observed GC-induced autophagy and progression features.

### miR-378 target genes are involved in autophagy and cancer progression

**Fig. 8:**
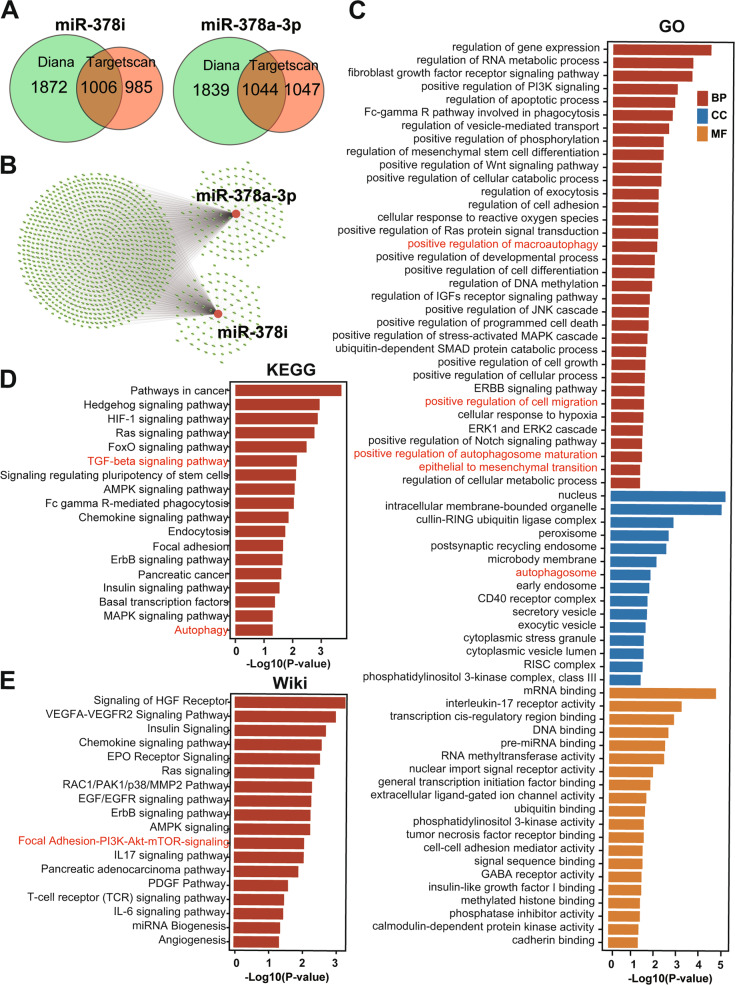
Detection of miR-378 target genes and related autophagy and cancer progression signaling via bioinformatics analysis. **A** Target genes of miR-378i and miR-378a-3p were predicted using the TargetScan (green) and DIANA-microT (red) databases and are shown as Venn plots. **B** The identified target genes were organized into a gene regulatory network using Cytoscape. Red circles: miR-378a-3p and miR-378i; red lines represent the connection of the specific miR to its corresponding target genes. To further highlight associations between miR-378i and miR-378a-3p target genes and signaling chains, the comprehensive gene set enrichment analysis web server Enrichr (https://maayanlab.cloud/Enrichr/enrich) was used to analyze gene sets in different databases. **C** Gene Ontology (GO) enrichment analysis was performed. BP biological process, CC cellular component, MF molecular function. **D** Likewise, Kyoto Encyclopedia of Genes and Genomes (KEGG) pathway analysis was conducted. **E** The WikiPathways database of biological pathways was screened.

These in vitro findings are supported by bioinformatics evaluation and the use of the TargetScan and DIANA databases. The number of overlapping genes was identified by Venn analysis, which identified 1872 and 995 target genes for miR-378i by DIANA and TargetScan analysis, respectively, with 1008 overlapping genes suggested by both databases (Fig. 8A). Likewise, DIANA identified 1839 and TargetScan identified 1047 target genes for miR-378a-3p, among which 1044 were overlapping genes. Next, a Cytoscape miRNA‒target gene regulatory network analysis was performed and demonstrated that miR-378i and miR-378a-3p target genes are highly overlapping (Fig. 8B), indicating that the biological functions of these miRs are quite similar. To understand the biological implications of miR-378 in more detail, online analysis was performed using Gene Ontology (GO), Kyoto Encyclopedia of Genes and Genomes (KEGG), and the Wiki pathway (Fig. 8C–E). The Enrichr database, a robust enrichment analysis online tool linked to mammalian gene set libraries and pathway databases [43], was used for analysis. The obtained results demonstrate that the key terms and pathways are involved in autophagy and progression features and thereby support our previous findings.

## Discussion

Here, we validated the concept of whether the described DEX-induced progression and therapy resistance of PDAC [7, 34] might involve miRNA-induced autophagy signaling. We identified pronounced features of autophagy upon treatment of established PDAC cell lines with DEX and various other GCs. Most importantly, we detected significantly enhanced expression of autophagy markers in the tissue of PDAC patients who had received GC medication prior to resection as compared to untreated PDAC control subjects. Mechanistically, we identified miR-378 and a complex network of target genes as regulators of DEX-induced autophagy and PDAC progression. We demonstrated that direct binding of miR-378 to the DEX-GR complex is most likely responsible for the observed signaling chains. Our comprehensive evaluation of GC-induced miR-378 signaling, which promotes autophagic flux and PDAC progression, provides novel knowledge of high clinical significance. In general, there are currently only a few publications which report GC-induced autophagy, and most of them are not in the context of cancer.

Consistent with our data, recent studies described that GC treatment of human chondrocytes induced autophagy and was cytoprotective; similarly, the inhibition of autophagy in malignant lymphoid cells was able to overcome GC resistance [20, 21]. Likewise, inhibition of serum- and glucocorticoid-inducible kinase 2 (SGK2) in epithelial ovarian cancer blocked autophagy and thereby sensitized cells to platinum drugs [44]. Additionally, our results are in line with the general assumption that PDAC exhibits elevated basal autophagy levels compared to other epithelial cancers and that autophagy plays multiple roles in the regulation of PDAC pathogenesis and treatment [45]. It is important to note that these elevated basal autophagy levels may be due to the extremely dense tumor stroma in PDAC, which is associated with low blood supply and high tumor hypoxia: these conditions are prerequisites for the induction of autophagy, the physiological function of which is to provide energy when both oxygen and glucose are depleted [22]. Consequently, elevated LC3 expression was found in 71 PDAC tissues, and strong LC3 expression correlated with significantly poor outcome, short disease-free period, tumor size, and tumor necrosis [22]. In the same way, autophagy is reported to protect the more aggressive pancreatic cancer stem-like cells from apoptosis induced by depletion of oxygen and nutrition [25].

Our results match these recent findings and demonstrate that GC medication significantly increased basal autophagy signaling, as we detected in 35 PDAC tissues from patients who received GC medication prior to surgery. Therefore, blocking autophagy may be envisaged as a therapeutic option in the treatment of PDAC, and this suggestion is supported by our results of lower autophagy levels upon treatment of PDAC cells with DEX in the presence of the GR inhibitor mifepristone/RU486. Similarly, blocking autophagy-related genes by siRNA transfection sensitized cell lines established from breast, cervical and lung cancer to radiation therapy [46]. Accordingly, Piffoux and colleagues suggested autophagy as a therapeutic target in cancer [47]. In particular, our data reveal that GC treatment further enhances the per se enhanced basal autophagy levels in PDAC and thereby furthers cancer progression. In fact, autophagy is considered to be a double-edged sword in cancer development and progression. Under normal growth conditions, basal autophagy contributes to cellular homeostasis by selective degradation of damaged or redundant organelles, which maintains genome integrity and prevents tumor growth [48]. However, excessive autophagic flux, as occurs in PDAC and which is further increased by GC treatment, converges into rapid tumor growth and progression.

Little is known about the underlying mechanisms of GC-induced autophagy in PDAC. Our results imply that DEX treatment mediates the accumulation of autophagic vacuoles, including autophagosomes and autolysosomes, converts LC3-I to LC3-II, and degrades SQSTM1/p62—these are typical features of macroautophagy [27]. Additionally, we observed that the enhanced autophagic flux upon DEX treatment was largely inhibited by the GR antagonist mifepristone/RU486, suggesting that binding of a GC to the GR is necessary for induction of autophagy. To address this assumption, we used computational modeling of RNA-small molecule interactions, that has become an indispensable bioinformatics tool for RNA-targeted drug discovery and detection of RNA docking to molecules [49–51]. Indeed, by performing these bioinformatics molecular docking studies, we found that binding of DEX-induced miR-378 to the GC-GR complex is more stable than binding of miR-378 to DEX alone. Our discovery confirms recent reports about a key function of miRNAs in GC signaling [41] as well as in the development of PDAC [52]. Therefore, to identify those miRNAs that are induced by GCs and subsequently involved in progression of PDAC, we performed microarray analysis, bioinformatics assessment and RT-qPCR and thereby detected 12 significant DEX upregulated miRNAs with the most significantly upregulated miR-378 top candidate, represented by its subtypes miR-378i and 378a-3p. We demonstrated variability of miR-378 expression between nonmalignant, low malignant and highly malignant cell lines and observed a correlation of the miR-378 expression level to the malignancy of the corresponding cell line. Thus, nonmalignant pancreatic cells or low-malignant PDAC cells had an almost low miR-378 expression. In contrast, highly-malignant MIA-PaCa2 and AsPC-1 PDAC cell lines had high miR-378 expression. These results are reflected by the overall survival of PDAC patients: high miR-378 expression correlates with shorter survival. This suggests an oncogenic function of miR-378, which can be induced by glucocorticoid treatment as reflected by enhanced miR-378 expression, autophagy, cancer stem cell characteristics, and cancer progression. Indeed, we were then able to block GC-induced autophagy and PDAC progression features by siRNA-mediated inhibition of miR-378.

In conclusion, this study provides data to support the conclusion that GCs enhance PDAC tumor progression by miR-378-mediated induction of autophagy. Moreover, our encouraging results obtained with inhibition of miR-378 or GR signaling suggest that both molecules are promising therapeutic targets in PDAC treatment. Considering that GCs are frequently described and that even endogenous GC levels are often enhanced due to severe stress, chronic pancreatitis, depression, protein-rich meals, and acute smoking [53], our findings provide further evidence of the adverse effects of GC administration in epithelial cancer therapy.

## Materials and methods

### Cell culture

The human PDAC cell lines BxPc-3, Panc-1, MIA-PaCa2, and AsPC-1 and the nonmalignant human pancreatic ductal cell line CRL-4023 (hTERT-HPNE-immortalized) were purchased from the American Type Culture Collection (ATCC, Manassas, VA, USA). Gemcitabine-resistant BxGEM cells were selected from parental BxPc-3 cells by continuous treatment with increasing concentrations of gemcitabine up to 200 nM for more than one year [54]. The human primary pancreatic cancer cell line ASAN-PaCa was kindly provided by Dr. N. Giese and is described [55]. BxPc-3, BxGEM, Panc-1, MIA-PaCa2, ASAN-PaCa, and AsPC-1 cells were cultured in Dulbecco’s modified Eagle’s medium with high glucose supplemented with 100 µg/ml fetal bovine serum (both from Sigma‒Aldrich, Taufkirchen, Germany) and 1 mM HEPES (PAA Laboratories, Posching, Austria). CRL-4023 cells were cultured in Dulbecco’s modified Eagle’s medium without glucose (ThermoFisher Scientific) and Medium M3 Base (Incell Corp, San Antonio, USA) at a ratio of 3:1 with 2 mM l-glutamine, adjusted to 1.5 g/l sodium bicarbonate, and supplemented with 5% fetal bovine serum, 10 ng/ml human recombinant EGF, 750 ng/ml puromycin (all from Sigma‒Aldrich), and 5.5 mM D-glucose (Merck, Taufkirchen, Germany). All cell lines were cultured at 37 °C in a humidified atmosphere of 95% O_2_ and 5% CO_2_ and were recently authenticated by SNP profiling (Multiplexion GmbH, Heidelberg, Germany) and by their typical morphology throughout the culture. To maintain the authenticity of the cell lines, frozen stocks were prepared from initial stocks, and every three months, a new frozen stock was used for the experiments. Mycoplasma-negative cultures were ensured by monthly testing by PlasmoTest™ (InvivoGen, San Diego, USA).

### Patient tissue

Tissue specimens were obtained from patients who had undergone surgery at the Department of General, Visceral and Transplant Surgery, University of Heidelberg, from January 2014 to June 2020. The Ethics Committee of the University of Heidelberg approved the study after receiving written informed consent from the patients. Clinical diagnoses were established by conventional clinical and histological criteria. Surgical resection was performed as indicated by the principles and practice of oncological therapy.

### Reagents

Stock solutions were prepared by dissolving DEX (25 mM, ≥98%) and mifepristone (RU486, 50 mM, ≥98%) in ethanol, cortisone (25 mM, ≥98%) in methanol, chloroquine (100 mM, ≥98.5%) in PBS, and bafilomycin A1 (100 μM, ≥90%), betamethasone (50 mM, ≥98%), budesonid (100 mM, ≥99%), and prednisone (100 mM, ≥98%) in DMSO (all reagents were from Sigma‒Aldrich, Munich, Germany). Rapamycin (20 mM, ≥98%, Axxora, Lörrach, Germany) was prepared in DMSO. Gemcitabine (126 mM, Lilly Deutschland, Bad Homburg, Germany) was freshly diluted in cell culture medium to a 100 μM stock solution. The final concentrations of the solvents in the media were 0.1% or less.

### MTT assay

A total of 3 × 10^3^ cells/100 µl per well of a 96-well plate was seeded. Twenty-four hours later, the cells were treated or left untreated as the control. After 24, 48 and 72 h of incubation, 10 µl MTT (Sigma‒Aldrich, Taufkirchen, Germany) was added to each well and incubated at 37 °C for 4 h until the formation of violet formazone crystals became visible. Then, 200 µl of DMSO was added to each well and incubated with gentle shaking at 37 °C for 5 min. The absorbance was measured at 570 nm using a FLUOstar OPTIMA microplate reader (BMG LABTECH, Ortenberg, Germany) with a reference wavelength of 630 nm.

### Transmission electron microscopy

The cells were seeded in 24-well plates containing microscope cover slides (Thermo Fisher, Saarbrucken, Germany) at a density of 3 × 10^4^/ml and in the presence of 1 µM DEX or ethanol solvent control for 24 h. Afterward, the cells were fixed with 2.5% glutaraldehyde (Sigma‒Aldrich, Taufkirchen, Germany) in 0.1 mM sodium-cacodylate buffer (Sigma‒Aldrich). The samples were stained with a negatively charged 1% aqueous phosphotungstic acid solution or a 2% acetate solution (both from Sigma‒Aldrich), which were dropped into a 200 μm mesh-size pioloform-coated copper grid or a microscope carbon-coated grid using a micropipette. The samples were analyzed under a Zeiss EM10 transmission electron microscope (Carl Zeiss, Oberkochen, Germany) at 80 kV and ×6000 magnification.

### miRNA transfection

The MirVana™ inhibitors hsa-miR-378i, hsa-miR-378a-3p and hsa-noncoding miRNA (miR-NC) were from Thermo Fisher Scientific (Dreieich, Germany). The miRs were reverse transfected at a concentration of 100 nM each using Lipofectamine RNAiMAX (Thermo Fisher) as described in the manufacturer’s instructions.

### Colony forming assay

Twenty-four hours after transfection, the cells were treated with 1 µM DEX or ethanol vehicle control for 24 h. Then, the cells were reseeded at a low density of 400 cells/well in six-well plates in triplicate, followed by incubation for 14 days without changing the cell culture medium. After fixing with 3.7% paraformaldehyde, staining with 0.05% Coomassie Blue, washing and drying overnight, the number of colonies comprising at least 50 cells was counted by microcopy. The number of colonies in the control was set to 1, and the plating efficiency was calculated as 100 × (number of colonies/number of seeded cells).

### Spheroid assay

For spheroid formation, the cells were cultured in NeuroCult NS-A basal serum-free medium for human cells (StemCell Technologies, Vancouver, Canada) supplemented with 2 μg/ml heparin (StemCell Technologies), 20 ng/ml hEGF (R&D Systems, Wiesbaden-Nordenstadt, Germany), 10 ng/ml hFGF-b (PeproTech, Hamburg, Germany) and NeuroCult NS-A Proliferation Supplements (StemCell Technologies). The cells were seeded at low densities (5 × 10^2^ cells/ml) in 12-well low-adhesion plates (1 ml/well) after transfection and treatment as described in the colony formation assay. Five days later, the percentage of viable spheroids of this first generation (1st Gen) was determined by setting the number of spheroids in the control to 100%. Thereafter, 1st Gen spheroids were dissociated into single cells, and equal numbers of live cells were replated at a concentration of 5 × 10^2^ cells/ml. Upon spheroid formation 5 days later, the cells were photographed at ×100 magnification and quantified as second generation (2nd Gen) spheroids as described above.

### Wound healing assay

Twenty-four hours after transfection, the cells were treated with 1 µM DEX or ethanol vehicle control (CO) for 24 h. Then, the cells were resuspended in DMEM supplemented with 10% FCS and plated at a high density of 90% confluence before being scratched in six-well tissue culture plates by the use of a 10-μl pipette tip, followed by replacement of the culture medium with serum-free DMEM. The wounded region was microscopically recorded at ×100 magnification immediately after scratching (0 h) and 24 h later. The percentage of the gap area at 24 h relative to the area at 0 h was evaluated by microscopy and ImageJ software (NIH, Bethesda, MD, USA).

### Transwell migration assay

The cells were transfected and treated as described above. Then, 1 × 10^5^ cells/well were seeded in 24-well plates with Transwell polycarbonate filters using 6.5 mm diameter inserts with 8 μm pores (Corning Life Sciences, Amsterdam, The Netherlands). The cells were cultured inside of each insert with 300 μl 1% FCS culture medium and in the lower well of the migration plate with either 500 μl 10% FCS, 1% FCS in the negative control or 20% FCS in the positive control. After 48 h of incubation, the cells were fixed with 4% paraformaldehyde, followed by staining with crystal violet. The nonmigratory cells on the interior of the insert were gently wiped off with a cotton swab. The migratory cells remained on the bottom of the insert membrane. The Transwell inserts were washed twice with PBS to remove unbound crystal violet and then air-dried. The migratory cells on the bottom of the insert membrane were examined microscopically at ×200 magnification. For analysis, crystal violet was eluted using 33% (v/v) acetic acid with dd H_2_O. The bound crystal violet was eluted by adding 400 μl of 33% acetic acid into each insert and shaking for 10 min, and 100 μl of the eluent was transferred to a 96-well plate and quantified by measuring the absorbance at 590 nm with a plate reader.

### MDC staining of autophagic vacuoles

Cells growing on coverslips were labeled with the autofluorescent agent monodansylcadaverine (MDC, Sigma‒Aldrich, Taufkirchen, Germany) by incubation with 0.05 mM MDC diluted in cell culture medium or PBS at 37 °C for 10-30 min in the dark, as described [56]. Then, the cells were washed 3× with PBS and immediately analyzed with a fluorescence microscope equipped with a V-2A excitation filter at 380/420 nm and a barrier filter at 450/525 nm. Images were captured using a SPOT™ FLEX 15.2 64MP shifting pixel digital color camera (Diagnostic Instruments, Sterling Heights, MI, USA). The MDC fluorescence intensity was calculated as integrated density [57], which means the sum of the values of the pixels in the image, in cells from three images using ImageJ software (NIH, Bethesda, MD, USA). The result was evaluated as the sum of MDC fluorescence intensity/number of cells. Positively stained cells in ten randomly selected fields were counted blindly by two examiners.

### Western blot analyses

The cells were lysed in RIPA lysis buffer (Abcam, Cambridge, UK), and total protein was isolated according to a standard protocol. The protein concentration was determined using the BCA Protein Assay Kit (Abcam, Cambridge, UK). Before SDS–PAGE separation, the samples were denatured by boiling for 5 min and then kept on ice. The separated proteins were transferred from the gel to a PVDF membrane using a semi-dry system. The membrane was blocked by incubation in 3% BSA solution and then incubated with primary antibodies, including a rabbit polyclonal antibody against LC3B (Cell Signaling Technology, Danvers, MA, USA, #2775), mouse monoclonal antibodies against SQSTM1/p62 (Cell Signaling Technology, Danvers, MA, USA, #5114), vimentin (ab V9, Abcam, Cambridge, UK, #ab8069), and β-actin (Sigma‒Aldrich, Munich, Germany, #A5441), and rabbit monoclonal antibodies against E-cadherin (ab 24E10, Cell Signaling Technology, Danvers, MA, USA, #3195) and GAPDH (Cell Signaling Technology, Danvers, MA, USA, #5174). After washing, the membranes were incubated with IRDye infrared dye-conjugated secondary antibodies (LI-COR Biosciences, Bad Homburg, Germany). The infrared intensity was measured using an Odyssey CLx Infrared Imaging System (LI-COR).

### microRNA microarray profiling

Total RNA was isolated with the miRNeasy Mini Isolation Kit (QIAGEN, Hilden, Germany) according to the manufacturer’s instructions. Microarray analyses were performed at the Genomics and Proteomics Core Facility of the German Cancer Research Center (DKFZ) Heidelberg using the Agilent Human miR Microarray (Release 19.0) covering 2006 human microRNAs. The raw array data were analyzed by the use of R software version 3.24.15 and the “limma” package. The Benjamini and Hochberg (BH) algorithm was used to correct for multiple testing and false discovery rate (FDR) [34]. The microarray data were uploaded to ArrayExpress under the accession number E-MTAB-12443.

### RT‒qPCR

Total RNA was isolated with the RNeasy Mini Kit (QIAGEN, Hilden, Germany). The RNA concentration was measured with a NanoDrop 2000 Spectrophotometer (NanoDrop Technologies, Wilmington, Delaware, USA), and 500 ng total RNA or miRNA was reverse transcribed to cDNA using the High-Capacity RNA-to-cDNA™ Kit, the TaqMan® microRNA Reverse Transcription Reagents kit, or the PowerUp™ SYBR™ Green Master Mix (all from Thermo Fisher Scientific, Hilden, Germany) according to the manufacturer’s instructions. Real-time PCR was performed using TaqMan Gene Expression master mix or TaqMan Universal PCR master mix and primers for hsa-miR-378i, hsa-miR-378a-3p, hsa-miR-210-3p, hsa-miR-200a-5p, hsa-miR-10a-3p, and RNU44 (all from Thermo Fisher Scientific). PCR was performed using a StepOne Real-Time PCR System (Thermo Fisher Scientific). Primer sequences for genes were provided by Origene (Herford, Germany) and Eurofins Genomics (Ebersberg, Germany) and are provided (Suppl. Table S5). PCR was conducted using 40 cycles of denaturation at 95 °C for 15 s, annealing at 56 °C for 15 s, and extension at 72 °C for 1 min. miRNA expression levels were normalized to the expression of RUN44. Gene expression levels were normalized to the expression of GAPDH. The fold gene expression indicated by all PCR results was calculated using the 2^−∆∆Ct^ method [58] in Excel.

### Detection of miR expression by in situ hybridization

The miRCURY LNA™ microRNA Detection Kit (QIAGEN, Hilden, Germany) was used to detect the expression of miRs in PDAC tissue sections according to the instructions of the manufacturer. The hybridization was performed for 2 h at 54 °C using a specific 3′,5′ digoxigenin (DIG)‑labeled LNA miR detection probe. A scrambled, DIG-labeled miR served as a negative control. The sequences of the miR probes were as follows: hsa-miR-378i: 5′-DiGN-CCTTCTGACTCCTAGTCCAGT-3′-DiGN_N; hsa-miR-378a-3p: 5′-DiGN-CCTTCTGACTCCAAGTCCAGT-3′-DiGN_N. The bound miR probes were detected with nitroblue tetrazolium/5-bromo-4-chloro-30-indolyl phosphate p-toluidine (NBT/BCIP; Vector Laboratories, Burlingame, CA, USA), which served as a substrate. For nuclear staining, patient tissue slices were incubated in Fast Red (Vector Laboratories, Burlingame, CA, USA). Positive signals in ten randomly selected fields were counted blindly by two examiners.

### Immunohistochemistry and immunofluorescence staining

Staining was performed on 6-µm frozen tissue sections as previously described [59]. The primary antibodies were mouse polyclonal against GR (Santa Cruz, Heidelberg, Germany, #sc-393232), rabbit monoclonal against LC3B (Abcam, Cambridge, UK, #ab48394), E-cadherin (ab 24E10, Cell Signaling Technology, Danvers, MA, USA, #3195), and mouse monoclonal against SQSTM1/p62 (Abcam, #ab56416), vimentin (ab V9, Abcam, Cambridge, UK, #ab8069). The positive signals were quantified using ImageJ software (NIH, Bethesda, MD, USA). Images of representative fields were captured using a SPOT™ FLEX 15.2 64MP shifting pixel digital color camera (Diagnostic Instruments) and analyzed with SPOT Basic/Advanced 4.6 software. Positively stained cells in ten randomly selected fields were counted blindly by two examiners.

### Bioinformatics docking analysis of miR binding to GR and DEX

The SDF file of the 3D structure of DEX (ID: 5743) was downloaded from PubChem (https://pubchem.ncbi.nih.gov/). The X-ray crystal structure of the GR receptor in complex with DEX (ID: 4UDC) was downloaded from the Protein Data Bank (PDB) database (https://www.rcsb.org/). The sequences of hsa-miR-378i and hsa-miR-378a-3p were downloaded from miRbase (https://www.mirbase.org/, version 22.1) and are “ACUGGACUAGGAGUCAGAAGG” and “ACUGGACUUGGAGUCAGAAGGC”, respectively. Interactions of DEX with miRNAs were calculated using a virtual screening module in YASARA Macro structure prediction software (http://www.yasara.org/macros.htm/, version 13.3.23). Based on the miRNA sequences, three-dimensional (3D) miRNA structures were constructed, followed by optimization. Likewise, the DEX 3D structure was optimized by Chem3D software (http://www.cambridgesoft.com/, version 14.0.0.117). The dock_run.mcr tool in YASARA Macro was applied to complete the global docking result analysis. The results of molecular docking were visualized using PyMol software (https://pymol.org/2/, version 2.4.0). Interactions of GR with DEX were calculated by applying a virtual screening module in the online ZDOCK server (https://zdock.umassmed.edu/version 3.0.2). First, the water molecules, metal ions, ligands, heteroatoms, and unwanted side chains in GR in complex with DEX were eliminated using PyMOL, and then the structure was optimized using YASARA Macro software. The same software was used to prepare a program database (PDB) file of the GR/DEX complex. The PDB file and 3D structures of hsa-miR-378i and hsa-miR-378a-3p were then uploaded to the online ZDOCK server. The docking results and hydrogen bonds were displayed by PyMOL software. “Energy minimization” was performed to find the lowest energy conformation of a molecule by YASARA Macro software. The hydrogen bonds between miR and DEX or miR and the GR/DEX complex in the binding site were determined and visualized using PyMol software.

### In silico analysis

The multiMiR (http://multimir.ucdenver.edu/) package in R software (V 4.1.0) was used to predict targets of miRNAs. Target genes were selected in both the TargetScan and DIANA-microT databases based on the top 70% among all conserved and nonconserved target sites. Overlapping target genes were detected with Venn diagrams using the R package “Venn”. A miRNA‒target gene network was constructed using Cytoscape software (https://cytoscape.org/, V 3.8.0), excluding cross-target genes from up- and downregulated miRNAs. To explore the biological implications of the target genes of miRNAs, the screened target genes were used for Kyoto Encyclopedia of Genes and Genomes (KEGG) pathway and Gene Ontology (GO) process analyses using the Enrichr database (https://maayanlab.cloud/Enrichr). The identified GO terms and KEGG and Wiki pathways were visualized by the use of the “dyply” package of R.

### Isolation of a primary PDAC cell line by tumor xenotransplantation, in vivo treatment, and reinjection into mice

The primary PDAC patient-derived xenograft line T30 was isolated from surgical specimens that were mechanically minced and transplanted into the flanks of NMRI (nu/nu) mice, followed by subtransplantation, as recently described [7]. Cancer spheres were isolated from T30 xenografts and cultured as spheroids in NeuroCult NS-A serum-free medium with supplements (STEMCELL Technologies, Cologne, Germany), followed by in vitro treatment with 1 μM DEX or vehicle control for 48 h. Equal amounts of 5 × 10^6^ viable cells in 100 μl PBS were then injected subcutaneously into the left and right flanks of 6-week-old NMRI (nu/nu) male mice (*n* = 6). After 3 weeks, the xenografts were resected, and autophagy-related marker proteins were determined by immunohistochemistry. The experiments were performed in the animal facilities of the University of Heidelberg after receiving approval from the authorities (Regierungspräsidium Karlsruhe, Karlsruhe, Germany). The investigator was blinded to the group allocation of the animals during the experiment. No statistical method was used to predetermine the sample size for the xenograft mice experiment, which was based on previous experimental observations. No data were excluded from the analysis.

### Overall survival analysis

The Kaplan‒Meier plotter online platform (http://kmplot.com/analysis/), which is an online database providing patient survival analysis, was used to analyze the expression levels of hsa-miRs 378i and 378a-3p in online available PDAC patient tissue and correlation with overall survival.

### Statistical analysis

In vitro experiments were performed in triplicate, and the data are expressed as the means ± SDs of at least 3 independent experiments. In vivo experiments were performed once in statistically relevant group sizes of 6 mice per group. The significance of the data was analyzed using Student’s *t* test for parametric data and the Kruskal–Wallis test and the Mann‒Whitney test with Bonferroni corrections for nonparametric data. SPSS 22.0 and JMP 14 software (Heidelberg, Germany) were used for statistical analysis, and *P* < 0.05 was considered statistically significant (**P < 0.01, *P < 0.05).

## Supplementary information

Crude Western blots

Supplementary Information

checklist

## Data Availability

The datasets supporting the conclusions of this article and its supplemental files are included within the article and thus are available.

## Code availability

The code for data cleaning and analysis associated with the current submission is available at https://github.com/MD-shan/Glucocorticoid. All R packages were loaded following the instructions for use.
