# Supplementary figures and images for: Glucocorticoid-induced microRNA-378 signaling mediates the progression of pancreatic cancer by enhancing autophagy
Source: Cell Death Dis. 2022 Dec 19;13(12):1052. doi: 10.1038/s41419-022-05503-3 (PMC9763328; doi:10.1038/s41419-022-05503-3)

**A**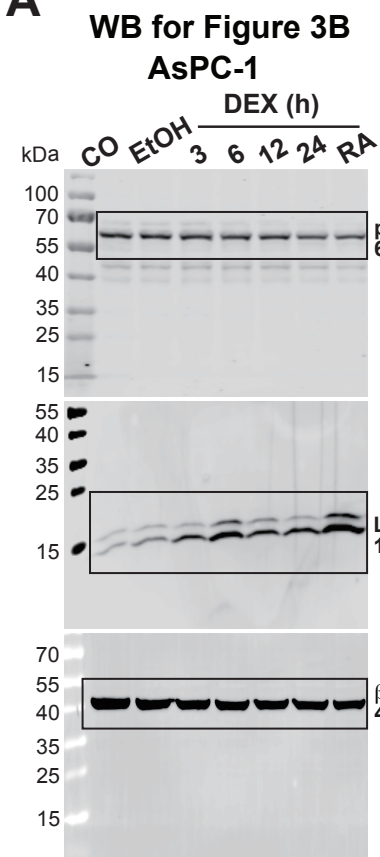**C**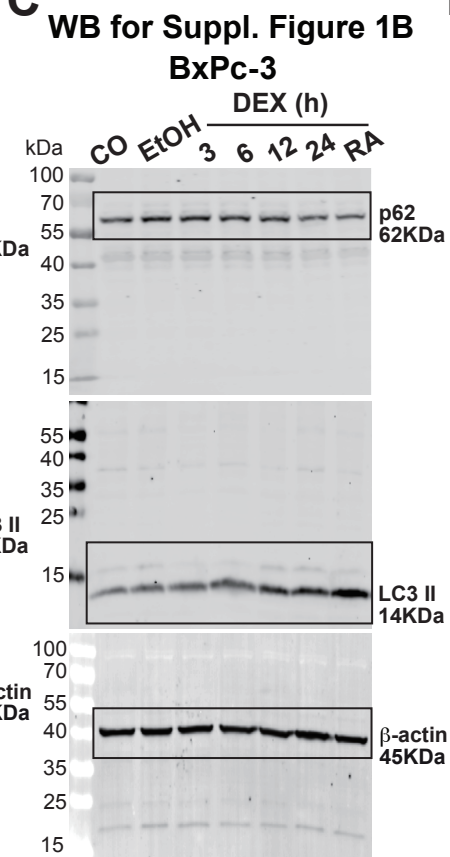**E**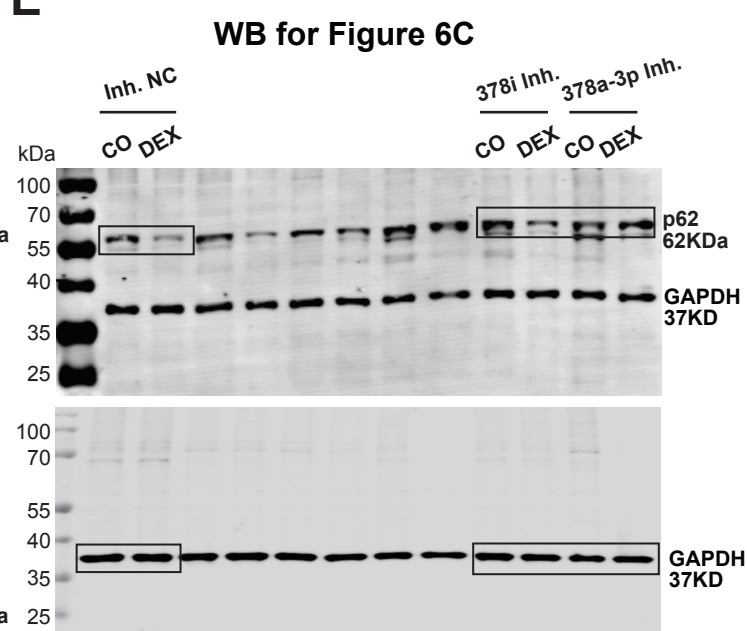**B**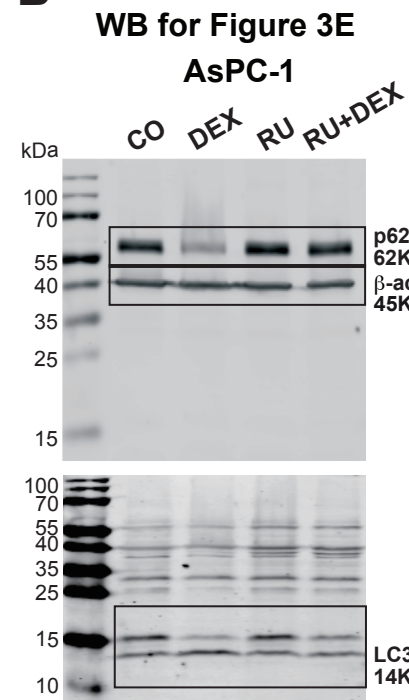**D**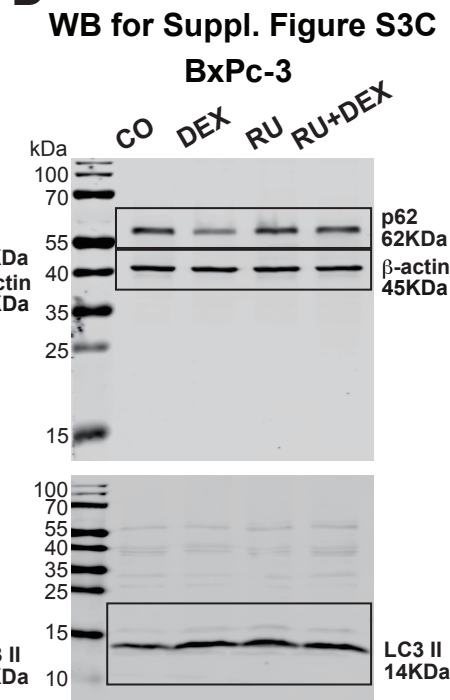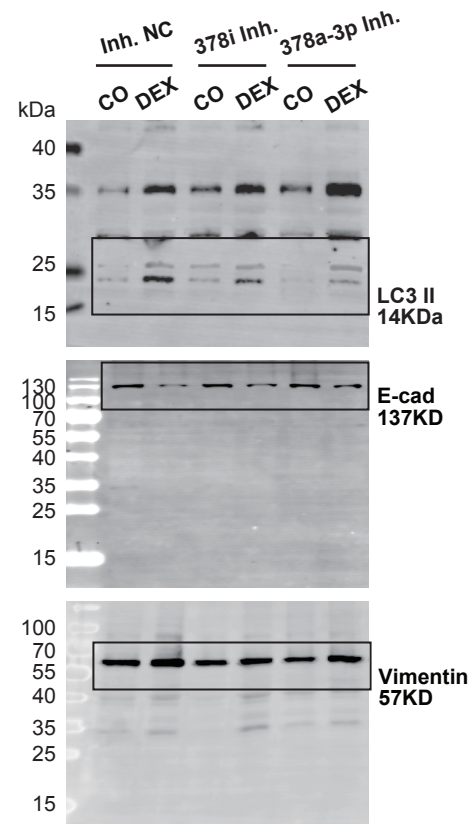

Supplement: Supplementary file 1 — Crude Western blots [file 41419_2022_5503_MOESM1_ESM.pdf]
