# Supplementary material for: Glucocorticoid-induced microRNA-378 signaling mediates the progression of pancreatic cancer by enhancing autophagy
Source: Cell Death Dis. 2022 Dec 19;13(12):1052. doi: 10.1038/s41419-022-05503-3 (PMC9763328; doi:10.1038/s41419-022-05503-3)
Supplement: Supplementary file 2 — Supplementary Information [file 41419_2022_5503_MOESM2_ESM.pdf]

Supplemental Information

Glucocorticoid-induced microRNA-378 signaling mediates the progression of pancreatic cancer by enhancing autophagy

Li Liu[1], Shanshan Han[1], Xi Xiao[1], Xuefeng An[1], Jury Gladkich[1], Ulf Hinz[2], Stefan Hillmer[3], Torsten Hoppe-Tichy[4], Yi Xu[5], Michael Schaefer[1], Oliver Strobel[2,6], Ingrid Herr[1,2#]

TABLE OF CONTENT

Supplemental Tables

Table. S1 Glucocorticoid intake by patients prior to pancreatic cancer resection. 2
Table S2 Patient information for pancreatic cancer tissues from the EPZ Pancobank 3
Table S3 miR candidates and known biological functions.. 4
Table S4 Hydrogen-bonding interaction sites of docked molecules 5
Table S5 Primer sequences.. 6

Supplemental Figures with Legends

Fig. S1 DEX treatment is associated with the expression of autophagy markers........ 7
Fig. S2 Crude western blot data 9
Fig. S3 The GR inhibitor RU486 inhibits DEX-induced autophagy 10
Fig. S4 Various GCs enhance basal viability and induce gemcitabine resistance..... 12
Fig. S5 miR-378 regulates DEX-induced autophagosome formation 13
Fig. S6 miR-378 regulates DEX-induced cancer stem characteristics 14

Table S1 Glucocorticoid intake by patients prior to pancreatic cancer resection

Patient ID	Corticoid Name	Dosis	Route of administration
5025-1A	Prednisolone	5 mg	orally
5082-2A	Beclometasone dipropion	100 µg	inhalatively
	Fluticasone furoate	27.5 µg	inhalatively
5174-1A	Fluticasone-17-propionat	250 µg	inhalatively
5266-1A	Fluticasone furoate	100 µg	inhalatively
5353-1A	Budesonide	3 mg	orally
5522-1A	Prednisolone	2 mg	orally
5676-1A	Beclometasone dipropion	100 µg	inhalatively
5746-1A	Prednisolone	5 mg	orally
5777-1A	Budesonide	200 µg	inhalatively
5783-1A	Prednisolone	20 mg	orally
5808-1A	Budesonide	200 µg	inhalatively
5833-2A	Budesonide	0.1 mg	inhalatively
5853-1A	Budesonide	0.2 mg	inhalatively
5858-1A	Budesonide	200 µg	inhalatively
5909-1E	Budesonide	0.2 mg	inhalatively
5936-1A	Prednisolone	5 mg	orally
5954-1A	Budesonide	400 µg	inhalatively
6093-1A	Beclometasone dipropion	100 µg	inhalatively
	Fluticasone furoate	27.5 µg	inhalatively
6133-1A	Prednisolone	20 mg	orally
6459-1A	Hydrocortisone	10 mg/g*	via the skin
6540-1A	Budesonide	200 µg	inhalatively
6726-1A	Prednisolone	5 mg	orally
6745-1A	Prednisolone	5 mg	orally
6843-1A	Prednisolone	10 mg	orally
6845-1A	Prednisolone	5 mg	orally
6847-1B	Budesonide	400 µg	inhalatively
7017-1B	Prednisolone	5 mg	orally
7020-3A	Fluticasonpropionate	250 µg	inhalatively
7068-1A	Prednisolone	5 mg	orally
7106-1A	Prednisolone	5 mg	orally
7696-1A	Beclometasone dipropionat	100 µg	inhalatively
7722-1A	Prednisolone	2 mg	orally
7889-1A	Prednisolone	1 mg	orally
7979-1A	Prednisolone	5 mg	orally
7982-1A	Beclometasone dipropionat	100 µg	inhalatively

*10 mg/g: 1 g ointment contains 10 mg hydrocortisone

Table S2 Patient information for pancreatic cancer tissues from the EPZ Pancobank

Intern No.	Tissue No.	Sex	Age	pT	pN	M	LN+	LNs examined	G	R	
5897	1	F	51	3	2	0	8	42	2	1	No GC treatment prior to resection
5913	2	M	58	2	1	0	1	17	2	1	
5921	3	F	67	2	1	0	2	25	2	1	
5934	4	F	52	3	1	0	1	53	3	1	
5956	5	F	53	3	1	0	1	37	2	1	
6026	6	F	74	3	2	0	4	36	3	1	
6034	7	F	58	3	0	0	0	40	2	1	
6040	8	F	69	3	1	0	2	35	3	1	
6041	9	M	51	3	1	0	1	38	3	1	
6053	10	M	51	2	2	0	7	42	2	1	
6055	11	M	57	2	1	0	1	21	2	1	
6065	12	M	79	2	1	0	1	36	2	1	
6069	13	M	66	2	1	0	1	18	3	1	
6071	14	M	76	3	1	0	7	37	2	1	
6073	15	F	50	3	1	0	3	34	2	1	
6074	16	F	88	3	1	0	3	31	2	1	
6076	17	F	80	3	1	0	2	32	2	0	
6095	18	F	49	3	1	0	2	35	3	1	
6102	19	F	73	3	2	0	5	38	3	1	
6111	20	M	73	3	2	0	4	42	3	1	
6113	21	M	76	3	2	0	11	41	3	1	
6121	22	F	79	2	2	0	5	37	3	1	
6122	23	F	63	3	1	0	3	13	2	0	
6127	24	F	56	3	2	0	4	47	2	1	
6137	25	F	87	3	1	0	2	24	2	1	
6143	26	F	77	2	2	0	5	31	2	1	
6144	27	F	55	2	2	0	10	31	3	1	
6169	28	F	64	2	2	0	11	25	2	1	
7097	29	M	56	2	1	0	3	33	2	1	
7105	30	F	75	2	2	0	5	28	2	1	
7114	31	M	76	1	0	0	0	17	3	1	
7115	32	F	63	3	2	0	7	25	2	1	
7131	33	F	80	2	2	0	9	38	3	1	
7132	34	F	44	1	1	0	2	39	3	0	
7152	35	M	70	3	2	0	9	42	2	1	
5025	1	M	72	2	2	0	16	16	3	1	GC treatment prior to resection
5082	2	F	51	2	1	0	1	34	3	1	
5174	3	F	75	3	1	0	2	51	2	1	
5266	4	F	66	2	1	0	1	28	3	1	
5353	5	F	54	2	2	0	8	32	2	1	
5522	6	F	79	3	1	0	1	36	2	1	
5676	7	M	65	2	0	0	0	39	3	1	
5746	8	F	67	1	0	0	0	18	2	0	
5777	9	M	68	1	0	0	0	31	3	1	
5783	10	F	74	3	2	0	18	26	2	1	
5808	11	M	56	2	0	0	0	21	2	1	
5833	12	F	63	2	1	0	1	25	2	1	
5853	13	M	85	2	0	0	0	23	3	1	
5858	14	F	75	2	1	0	1	43	2	1	
5909	15	F	42	2	1	1	3	23	2	1	
5936	16	M	76	2	1	0	2	24	2	1	
5954	17	F	74	1	0	0	0	36	3	0	
6093	18	M	70	3	1	0	2	29	2	0	
6133	19	M	76	3	0	0	0	25	2	0	
6459	20	M	49	3	2	0	11	26	4	1	
6540	21	M	64	2	2	0	15	34	2	1	
6726	22	M	79	2	1	0	2	32	3	1	

6745	23	F	64	1	2	0	13	39	2	1
6843	24	M	68	2	2	0	4	42	3	1
6845	25	M	66	1	2	0	16	48	2	0
6847	26	M	65	3	1	1	26	53	3	1
7017	27	M	56	2	1	0	1	49		0
7020	28	F	77	2	1	0	2	30	3	1
7068	29	F	75	3	0	0	0	31	3	1
7106	30	M	74	3	2	0	6	20	3	1
7696	31	F	74	2	2	0	9	22	3	1
7722	32	F	73	2	2	0	12	36	2	0
7889	33	F	66	2	1	0	1	17	2	1
7979	34	M	78	3	2	0	11	29	3	1
7982	35	M	56	3	2	0	26	46	2	1

No.: Number; **F:** Female; **M:** Male; **GCs:** glucocorticoids. **pT1**: Tumor limited to the pancreas, 2 cm or less in greatest dimension; **pT2**: Tumor limited to the pancreas, more than 2 cm but less than 4 cm in greatest dimension; p**T3:** Tumor is than 4 cm in greatest dimension; **N0:** No regional lymph node metastasis; p**N1:** Metastasis in 1 - 3 regional lymph nodes; p**N2:** Metastasis in 4 or more regional lymph nodes; **M0:** No distant metastasis; **M1:** Distant metastasis; **LN+:** Lymph-node metastasis; **G2:** Moderately differentiated; **G3:** Poorly differentiated; **G4:** Undifferentiated; **R0:** Indicates the removal of all macroscopic visible tumor tissue, and the microscopic examination of margins reveals that there is no tumor tissue. **R1:** Indicates the removal of all macroscopic visible tumor tissue, but the microscopic examination of margins reveals that there is tumor tissue. **pT** and **pN** were restaged according to the 8th edition of the Union for International Cancer Control (UICC) staging system.[1]

References

1. Brierley JD, Gospodarowicz MK, Wittekind C. Union for International Cancer Control. TNM classification of malignant tumours. Eighth edition. John Wiley & Sons. (274 pages).

Table S3 miRNA candidates and known biological functions

miRNA	Expression	miRNA Function	Ref.
378	Upregulated	Promotion of autophagy; Inhibition of apoptosis	1
		Promotion of stemness and EMT	2
		Promotion of cell survival, tumor growth, and angiogenesis	3
		Regulator of oxidative energy metabolism, tumor progression and Warburg effect	4
		Regulator of lipid, glucose and energy metabolism, and mitochondrial dysfunction	5, 6
		Oncogenic function	7, 8
		Enhancement of stem cell properties	8
		Enhancement of cell survival, colony formation, and chemoresistance	9
378i	Upregulated	Induction of cancer invasion	10
378a-3p	Upregulated	Oncogenic function	11, 12
		Promotion of stemness and chemoresistance	13
		Inhibition of Akt/mTOR pathway and induction of LC3B	14

References

1. Li Y, Jiang J, Liu W, Wang H, Zhao L, Liu S, *et al.* microRNA-378 promotes autophagy and inhibits apoptosis in skeletal muscle. *Proc Natl Acad Sci U S A* 2018, **115**(46)**:** E10849-E10858.
2. Bayraktar R, Van Roosbroeck K, Calin GA. Cell-to-cell communication: microRNAs as hormones. *Mol Oncol* 2017, **11**(12)**:** 1673-1686.
3. Lee DY, Deng Z, Wang CH, Yang BB. MicroRNA-378 promotes cell survival, tumor growth, and angiogenesis by targeting SuFu and Fus-1 expression. *Proc Natl Acad Sci U S A* 2007, **104**(51)**:** 20350-20355.
4. Eichner LJ, Perry MC, Dufour CR, Bertos N, Park M, St-Pierre J, *et al.* miR-378(*) mediates metabolic shift in breast cancer cells via the PGC-1beta/ERRgamma transcriptional pathway. *Cell Metab* 2010, **12**(4)**:** 352-361.
5. Liu W, Cao H, Ye C, Chang C, Lu M, Jing Y, *et al.* Hepatic miR-378 targets p110alpha and controls glucose and lipid homeostasis by modulating hepatic insulin signalling. *Nat Commun* 2014, **5:** 5684.
6. Zhang Y, Li C, Li H, Song Y, Zhao Y, Zhai L, *et al.* miR-378 Activates the Pyruvate-PEP Futile Cycle and Enhances Lipolysis to Ameliorate Obesity in Mice. *EBioMedicine* 2016, **5:** 93-104.
7. Feng M, Li Z, Aau M, Wong CH, Yang X, Yu Q. Myc/miR-378/TOB2/cyclin D1 functional module regulates oncogenic transformation. *Oncogene* 2011, **30**(19)**:** 2242-2251.
8. Ma J, Wu D, Yi J, Yi Y, Zhu X, Qiu H, *et al.* MiR-378 promoted cell proliferation and inhibited apoptosis by enhanced stem cell properties in chronic myeloid leukemia K562 cells. *Biomed Pharmacother* 2019, **112:** 108623.
9. Wu QP, Xie YZ, Deng Z, Li XM, Yang W, Jiao CW, *et al.* Ergosterol peroxide isolated from Ganoderma lucidum abolishes microRNA miR-378-mediated tumor cells on chemoresistance. *PLoS One* 2012, **7**(8)**:** e44579.
10. Sugai T, Sugimoto R, Eizuka M, Osakabe M, Yamada S, Yanagawa N, *et al.* Comprehensive Analysis of microRNA Expression During the Progression of Colorectal Tumors. *Dig Dis*

Sci 2022.

11. Niu F, Dzikiewicz-Krawczyk A, Koerts J, de Jong D, Wijenberg L, Fernandez Hernandez M, *et al.* MiR-378a-3p Is Critical for Burkitt Lymphoma Cell Growth. *Cancers (Basel)* 2020, **12**(12).
12. Zhang Y, Yu R, Li L. LINC00641 hinders the progression of cervical cancer by targeting miR-378a-3p/CPEB3. *J Gene Med* 2020, **22**(9)**:** e3212.
13. Yang Q, Zhao S, Shi Z, Cao L, Liu J, Pan T, *et al.* Chemotherapy-elicited exosomal miR-378a-3p and miR-378d promote breast cancer stemness and chemoresistance via the activation of EZH2/STAT3 signaling. *J Exp Clin Cancer Res* 2021, **40**(1)**:** 120.
14. Jiao Y, Li W, Wang W, Tong X, Xia R, Fan J, *et al.* Platelet-derived exosomes promote neutrophil extracellular trap formation during septic shock. *Crit Care* 2020, **24**(1)**:** 380.

Table S4 Hydrogen-bonding interaction sites of docked molecules

miRNAs	Binding sites		Binding sites	
name	miRNAs	DEX	miRNAs	Complex
378i	G-4	O-4	C-2	TRP-712, HIS-775, PHE-774
			U-3	GLN-776
378a-3p	C-4	O-2	U-8	SER-708
			C-7	SER-708
			U-3	AGR-714
			G-11	TYR-716, HIS-775
			G-10	HIS-775

G: Guanine; C: Cytosine; U: Uracil; O: Oxygen atom; TRP: Tryptophane; HIS: Histidine; PHE: Alanine; GLN: Glutamine; SER: Serine; AGR: Arginine; TYR: Tyrosine.

Table S5 Primer sequences

Primer Name	Primer sequence 5` > 3`
LC3B forward	GAG AAG CAG CTT CCT GTT CTG G
LC3B reverse	GTG TCC GTT CAC CAA CAG GAA G
SQSTM1/p62 forward	TGT GTA GCG TCT GCG AGG GAA A
SQSTM1/p62 reverse	AGT GTC CGT GTT TCA CCT TCC G
ATG12 forward	GCT AAA GGC TGT GGG AGA CA
ATG12 reverse	TTG GAT GGT TCG TGT TCG CT
ATG4B forward	ATG GGA GTT GGC GAA GGC AAG T
ATG4B reverse	AGC TCC ACG TAT CGA AGA CAG C
GABARAPL1 forward	TTG TAG AGA AGG CTC CAA AAG CC
GABARAPL1 reverse	GGT CTC AGG TGG ATT CTC TTC C
BNIP3 forward	TCA GCA TGA GGA ACA CGA GCG T
BNIP3 reverse	GAG GTT GTC AGA CGC CTT CCA A
VPS34 forward	GCG TTC TTT GCT GGC TGC ACA A
VPS34 reverse	CTC CAA GCA ATG CCT GTA GTC TC
CATHEPSIN L forward	GAA AGG CTA CGT GAC TCC TGT G
CATHEPSIN L reverse	CCA GAT TCT GCT CAC TCA GTG AG
BCL2L2 forward	CAA GGA GAT GGA ACC ACT GGT G
BCL2L2 reverse	CCG TAT AGA GCT GTG AAC TCC G
ATG16L2 forward	GAG CAG CGA TAC CAG ATC ATC C
ATG16L2 reverse	CAG CAT TGA CCT CAG AGA GGT G
GAPDH forward:	GTC TCC TCT GAC TTC AAC AGC G
GAPDH reverse	ACC ACC CTG TTG CTG TAG CCA A

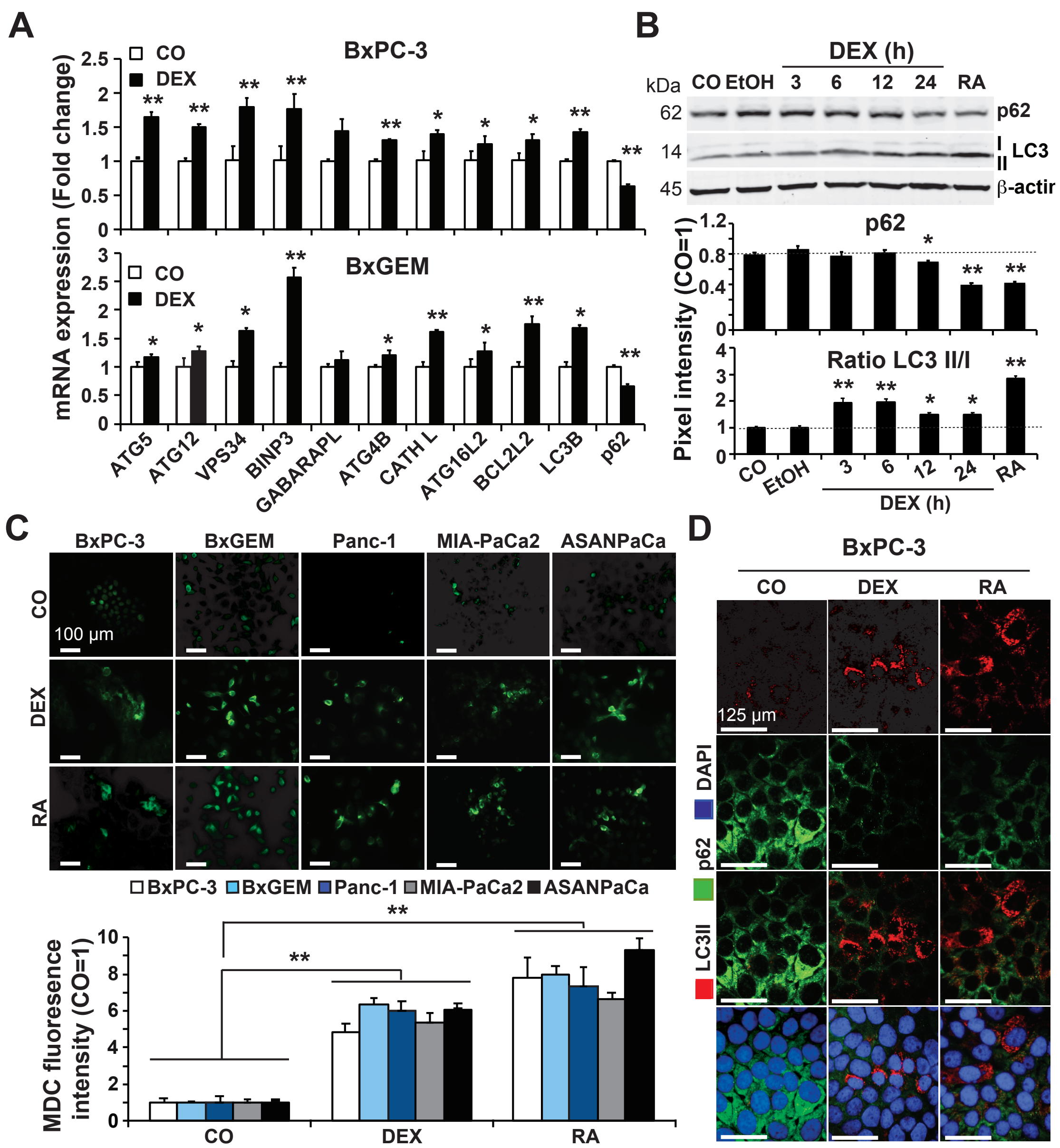

Fig. S1. DEX treatment is a ssociated with the expression of autophagy markers. (A) BxPC-3 and BxGEM cells were treated with 1 μM DEX or with ethanol vehicle control (CO), followed by harvesting of the total RNA 24 h later. RT–qPCR was performed using the specific autophagy-related primers ATG5, ATG12, VPS34, BINP3, GABARAPL, ATG4B, CATHEPSIN L (CATH L), ATG16L2, BCL2L2, LC3B, and SQSTM1/p62 (p62). Gene expression was normalized to that of GAPDH, and the relative mRNA expression is shown as the fold change. The data are presented as the mean values with standard deviations. **P*<0.05, ***P*<0.01 compared to the control group. **(B)** BxPC-3 cells were incubated in medium with 1 μM DEX and 20 μM rapamycin (RA), were left untreated (CO) or were treated with ethanol vehicle control (EtOH) for 24 h or the time points indicated. The expression of LC3-I, LC3-II and SQSTM1/p62 (p62) was detected by western blot analysis. β-Actin served as a loading control. The protein sizes in kilodalton (kDa) are provided on the left. The diagrams depict the pixel intensity of the bands, which was measured using Image Studio after normalization to β-actin. The pixel intensity of the control group was set to 1. ***P*<0.01

compared to the control group. The black dotted line represents 100%. **(C)** MDC staining of acidic vacuoles: BxPC-3, BxGEM, and ASAN-PaCa cells were cultured in medium with 1 μM DEX, 1 μM of the GR inhibitor mifepristone/RU486 (RU), DEX plus mifepristone/RU486 (DEX+RU), or were treated with ethanol vehicle control alone (CO) for 24 h. Autophagic vacuoles were labeled with the specific autofluorescence marker MDC (green), followed by immediate examination by fluorescence microscopy under 200× magnification. The scale bar indicates 100 μm. The MDC fluorescence intensity was quantified by ImageJ, and the density of the control was set to 1. **P*<0.05, ***P*<0.01. **(D)** BxPC-3 cells were treated with DEX or rapamycin (RA) alone as described above. Autophagy-related protein expression of LC3-II (red) and SQSTM1/p62 (p62, green) was detected by double-fluorescence staining under 1,000× magnification. The cell nuclei were counterstained with DAPI (blue).

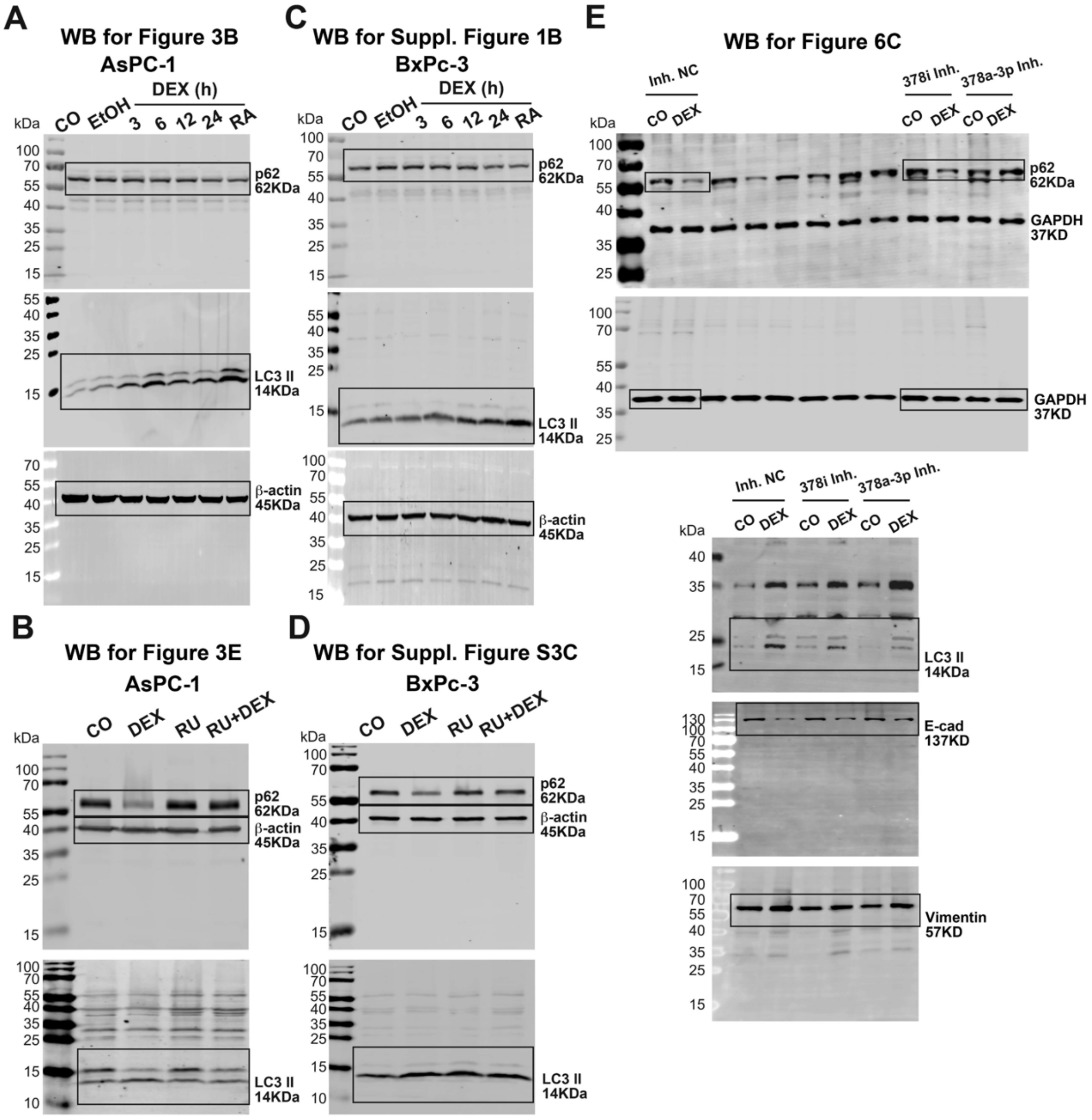

Fig. S2. Crude western blot data.

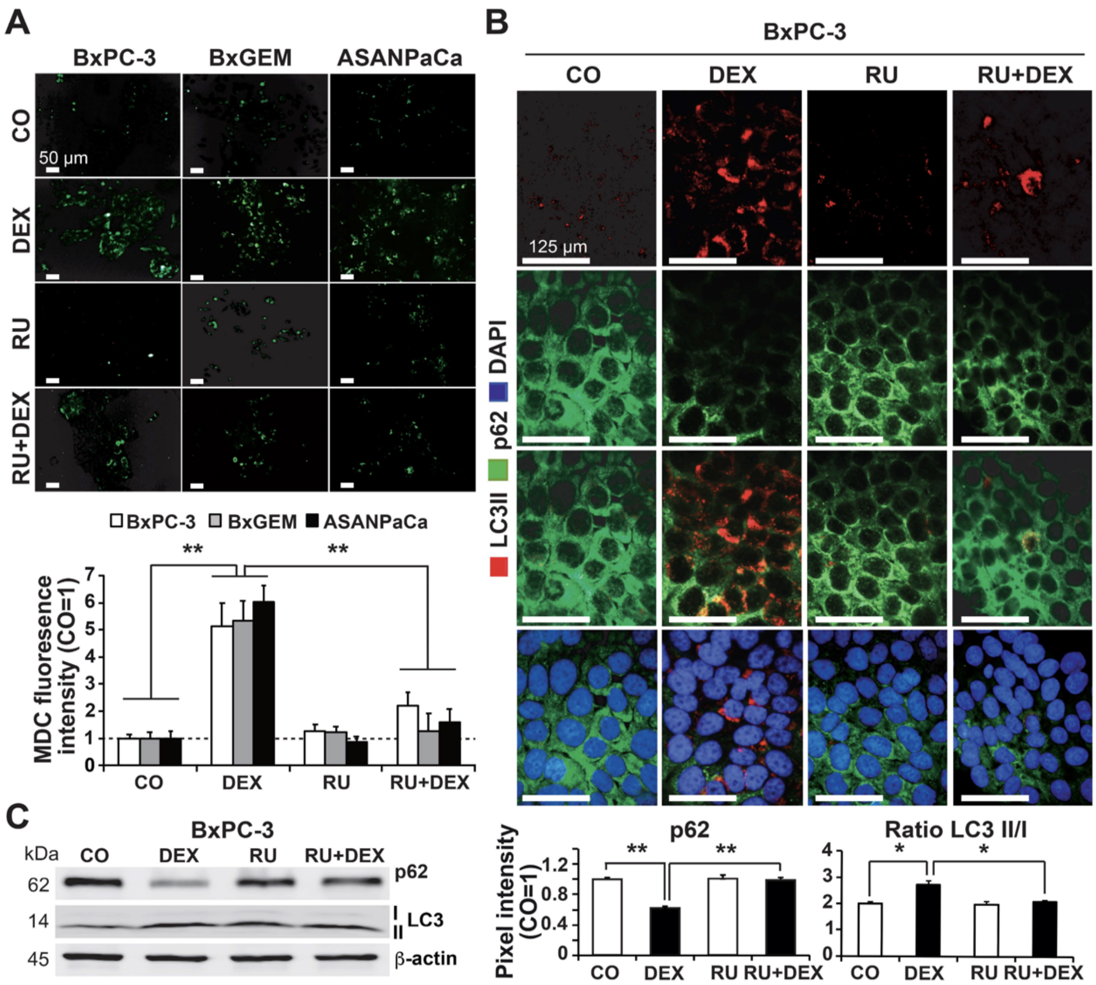

Fig. S3. The GR inhibitor RU486 inhibits DEX-induced autophagy. (A) MDC staining of acidic vacuoles: BxPC-3, BxGEM, and ASAN-PaCa cells were cultured in medium with 1 µM DEX, 1 µM of the GR inhibitor mifepristone/RU486 (RU), DEX plus mifepristone/RU486 (DEX+RU), or were treated with ethanol vehicle control alone (CO) for 24 h. Autophagic vacuoles were labeled with the specific autofluorescence marker MDC (green), followed by immediate examination by fluorescence microscopy under 200× magnification. The scale bar indicates 100 µm. The MDC fluorescence intensity was quantified by ImageJ, and the density of the control was set to 1. *P<0.05, **P<0.01. **(B)** BxPC-3 cells were cultured in medium with 1 µM DEX, 1 µM of the GR inhibitor mifepristone/RU486 (RU), DEX plus mifepristone/RU486 (DEX+RU), or were treated with ethanol vehicle control alone (CO) for 24 h. Autophagy-related protein expression of LC3-II (red) and SQSTM1/p62 (p62, green) was detected by double-fluorescence staining under 1,000× magnification. The cell nuclei were counterstained with DAPI (blue). The scale bar indicates 125 µm. **(C)** BxPC-3 cells were cultured in medium with 1 µM DEX, 1 µM mifepristone/RU486 (RU), both together (DEX+RU), or with ethanol vehicle control alone (CO) for 24 h. The expression of LC3-I, LC3-II and SQSTM1/p62 (p62) was detected by western blot analysis. β-actin served as a loading control. The protein sizes in kilodalton (kDa) are provided on the left. The pixel intensity of the bands was detected by the use of ImageJ, and the means and standard deviations are shown in the diagram. *P<0.05, **P<0.01.

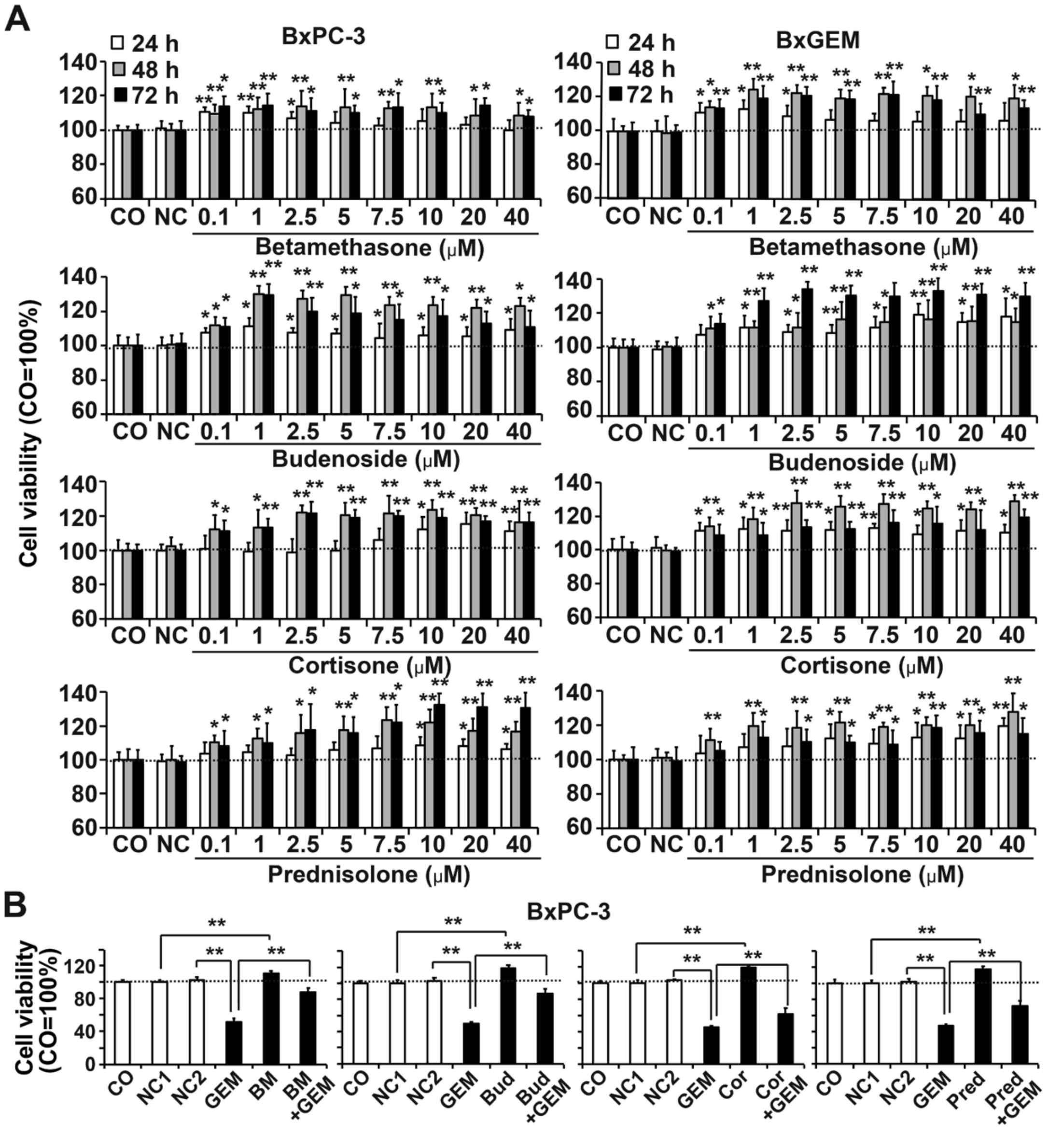

Fig. S4. Various GCs enhance basal viability and induce gemcitabine resistance. **(A)** BxPC-3 and BxGEM cells were treated with the GCs betamethasone, budesonide, cortisone, and prednisone at the concentrations indicated or with vehicle controls alone (NC1: DMSO 1:50,000; NC2: DMSO: 1:100,000; NC3: methanol: 1:25,000; NC4: DMSO: 1:100,000), followed by MTT assay 24 h, 48 h and 72 h later. The controls of each timepoint were set to 100%. *P<0.05, **P<0.01 compared to the negative control (NC). Cells were detected by MTT assay at 24 h, 48 h and 72 h after treatment as described in Fig. 5A. The data obtained with the controls of each timepoint were set to 100%. *P<0.05, **P<0.01 compared to the control group. **(B)** BxPC-3 cells were cultured in medium containing 1 µM betamethasone (BM), 1 µM budesonide (Bud), 10 µM cortisone (Cor), or 10 µM prednisone (Pred) or were treated with vehicle alone as described above (NC5: PBS: 1:126,000) or were left untreated (CO). Then, 48 h later, 50 nM gemcitabine (GEM) was added to untreated and GC-treated cells as indicated for another 48 h. The viability was measured by MTT assay. The percentage of viable cells in the control groups was set to 100%. *P<0.05, **P<0.01.

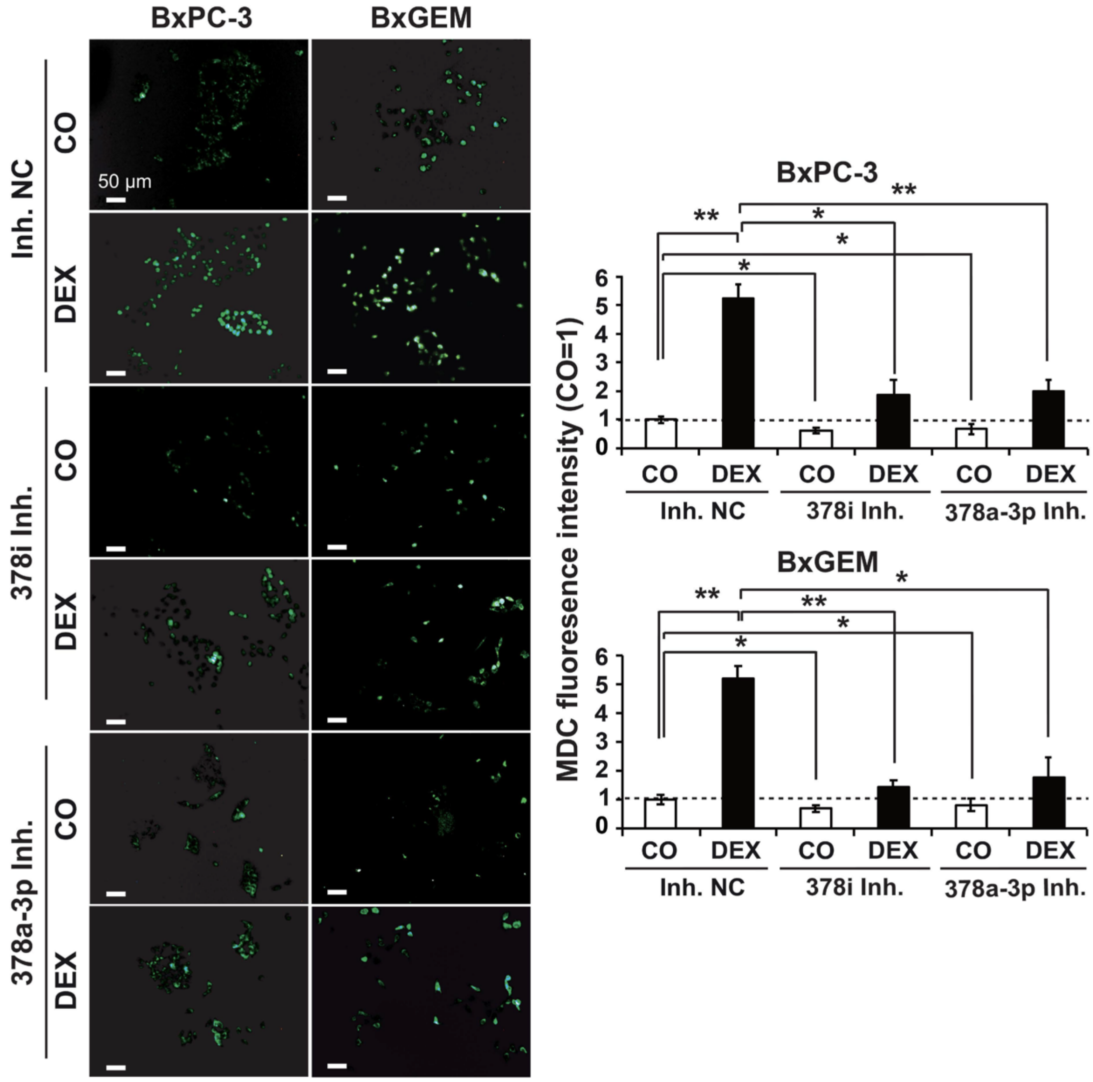

Fig. S5. miR-378 regulates DEX-induced autophagosome formation. BxPc-3 and BxGEM cells were lipotransfected with miR-378i inhibitor (100 nM), miR-378a-3p inhibitor (100 nM) or a noncoding miR inhibitor control (Inh. NC, 100 nM) and cotransfected with firefly luciferase (0.25 ng/µl), which served as a control for equal conditions. Twenty-four hours later, the cells were treated with 1 µM DEX or were left untreated as the control (CO). Then, 24 h later, autophagic vacuoles were labeled with the specific autofluorescence marker MDC (green), followed by immediate examination by fluorescence microscopy under 200× magnification. The scale bar indicates 50 µm. The MDC fluorescence intensity was quantified by ImageJ, and the density of the control was set to 1. **P*<0.05, ***P*<0.01.

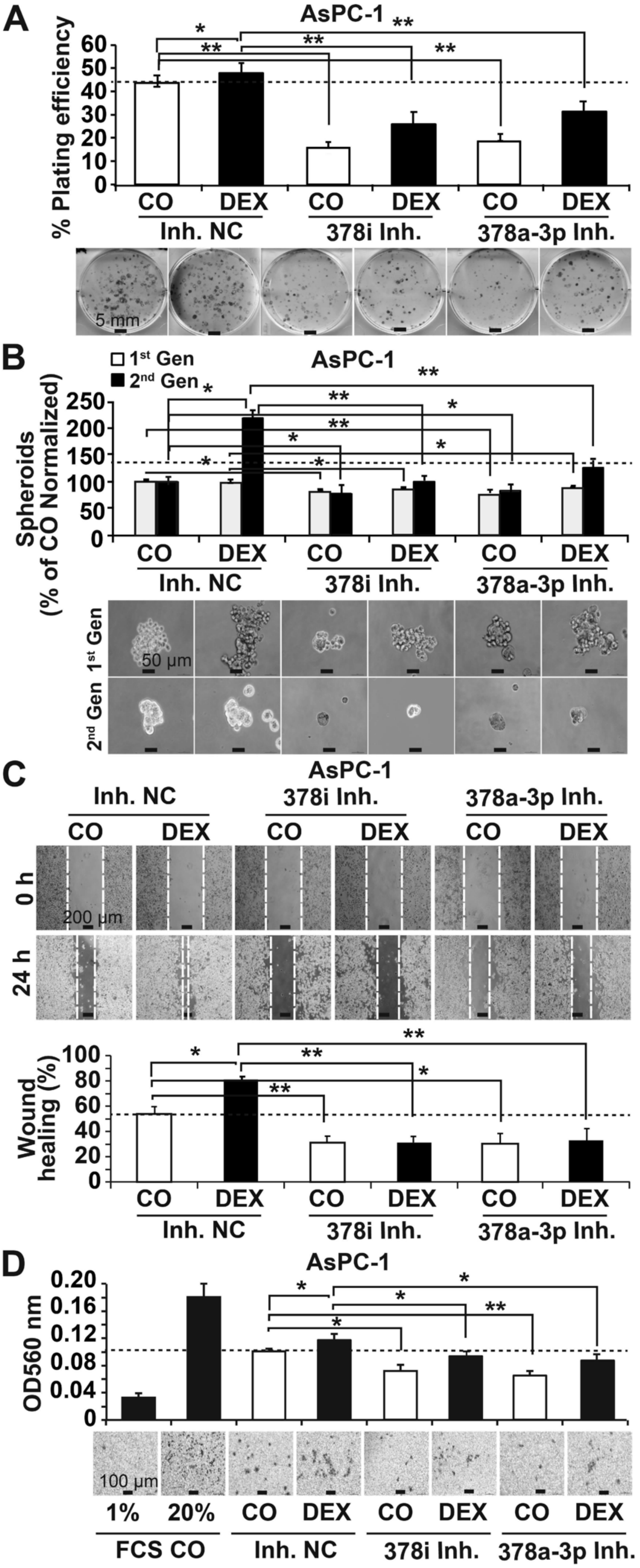
A
AsPC-1
% Plating efficiency
CO
DEX
Inh. NC
378i Inh.
378a-3p Inh.
5 mm
B
1st Gen
2nd Gen
Spheroids (% of CO Normalized)
50 µm
C
0 h
24 h
200 µm
Wound healing (%)
D
OD560 nm
100 µm
1%
20%
FCS CO

Fig. S6. miR-378 regulates DEX-induced cancer stem characteristics. (A) AsPC-1 cells were lipotransfected as described in Fig. S4. Twenty-four hours later, the cells were treated with 1 µM DEX or were left untreated as the control (CO). Then, 24 h later, the cells were resuspended in DMEM supplemented with 10% FCS and plated at a density of 400 cells/well in 6-well tissue culture plates. The cultures were maintained under standard culture conditions for two weeks followed by evaluation of 4% PFA fixed and 0.05% Coomassie-stained colonies consisting of at least 50 cells. The percentage of plating efficiency was calculated (number of colonies/number of seeded cells ×100). **(B)** Likewise, the cells were seeded at a low density of 5×10^2 cells/ml in ultralow attachment plates with serum-free but growth factor-containing medium for spheroid formation. Five days later, the percentage of viable spheroids of these so-called first-generation spheroids was determined (1st Gen). Thereafter, 1st Gen spheroids were dissociated into single cells, and equal numbers of live cells were replated at a concentration of 5×10^2 cells/ml and allowed to form second-generation spheroids (2nd Gen). Upon spheroid formation 5 days later, the cells were photographed at 100× magnification and quantified as described above. **(C)** Similarly, the cells were resuspended in DMEM supplemented with 10% FCS and plated at a high density of 90% confluence before scratching in 6-well tissue culture plates by the use of a 10-µl pipette tip. To stop proliferation, the medium was changed, and serum-free DMEM was added. The wounded region was microscopically recorded at 100× magnification immediately after scratching (0 h) and 24 h later (24 h). The percentage of the gap area relative to the original area was analyzed by ImageJ (http://imagej.net/Downloads, RRID:SCR_003070). **(D)** Finally, the cells were seeded at a low concentration of 1×10^5 cells per well into the upper chamber of Transwell plates in 1% FCS DMEM. The migration of the cells toward DMEM with 10% FCS in the lower chamber was evaluated. The migration of the cells toward DMEM with 1% FCS in the lower chamber served as a negative control. Migration toward DMEM with 20% FCS in the lower chamber served as a positive control. After incubation for 48 h, the cells were fixed with 4% paraformaldehyde, followed by staining with crystal violet. The cells on the upper membrane of the chamber were wiped off with a cotton swab, whereas the cells on the bottom membrane of the chamber were examined microscopically at 200× magnification. For analysis, the eluent of the migratory cells was transferred to a 96-well plate and quantified by measuring the absorbance at 590 nm. All of the above experiments were performed in triplicate, and identical outcomes and standard deviations are shown. *P<0.05, **P<0.01. The black dotted line represents the value of the control group.
